# Optimising acute toxicity monitoring in prostate MR-guided radiotherapy workflow: Results from a prospective study using multiple electronic PRO assessments

**DOI:** 10.1016/j.tipsro.2025.100368

**Published:** 2025-12-10

**Authors:** Pia Krause Møller, Helle Pappot, Tine Schytte, Uffe Bernchou, Karin Brochstedt Dieperink

**Affiliations:** a Department of Oncology, Odense University Hospital, J.B. Winsløws Vej 4, Odense C 5000, Denmark; b Department of Clinical Research, University of Southern Denmark, Campusvej 55, Odense M 5230, Denmark; c AgeCare, Academy of Geriatric Cancer Research, Odense University Hospital, Kløvervænget 9, Odense C 5000, Denmark; d Department of Oncology, Rigshospitalet, University Hospital of Copenhagen, Blegdamsvej 9, Copenhagen 2100, Denmark; e Department of Clinical Medicine, University of Copenhagen, Blegdamsvej 3B, Copenhagen 2200, Denmark; f Laboratory of Radiation Physics, Odense University Hospital, Kløvervænget 9, Odense C 5000, Denmark

**Keywords:** MRgRT, MR-linac, Symptom trajectory, Patient-reported outcomes, ePRO, Prostate cancer

## Abstract

**Introduction:**

Frequent electronic patient-reported outcomes (ePROs) may become an important tool for monitoring outcomes in ultrahypofractionated MRgRT for prostate cancer, enabling real-time remote tracking of toxicity. By integrating weekly ePROs and health-related quality of life (HRQoL) assessments into the early prostate MRgRT workflow, this study aimed to explore real-time acute symptom trajectories and the impact on HRQoL.

**Methods:**

Two cohorts were followed: Patients receiving MRgRT for localised PCa (60Gy/20fx) or low-volume metastatic (M1) PCa (36Gy/6fx) and eligible to complete weekly ePROs and HRQoL measures (EQ-5D-5L, EORTC QLQ-C30) during and up to 24 weeks of follow-up. Linear mixed models were used to evaluate symptom changes over time.

**Results:**

Of 76 included PCa patients, 42 had localised PCa and 34 low-volume M1 PCa. The linear model revealed significant changes in urinary symptoms from treatment week one, persisting 2 weeks post-MRgRT in the 36 Gy cohort, and 3–4 weeks in the 60 Gy cohort. Bowel symptoms increased early post-treatment in both cohorts, with diarrhoea being most frequent. Clinically relevant changes in HRQoL were observed during follow-up: patients in the 60 Gy cohort showed HRQoL improvements after 12 and 24 weeks. In the 36 Gy cohort, patients reported improved self-rated Global health status/QoL and emotional functioning.

**Conclusion:**

Frequent ePROs during and after MRgRT provide critical insights into the timing, fluctuation and severity of acute toxicity, potentially missed with standard follow-up schedules. Integrating real-time ePROs into the MRgRT workflow is a feasible patient-centered method to systematically optimize the outcome assessments of MRgRT.

## Highlights

• Multiple ePRO assessments reveal timely changes in the prevalence of acute toxicity.
• ePRO trajectories highlight symptom worsening outside standard follow-up schedules.
• Symptom peaks post-treatment underscore the need for frequent ePRO monitoring.
• Moderate symptom worsening did not translate into decreased HRQoL.
• High response rates of multiple ePROs among patients with localised and metastatic PCa.

## Introduction

As clinical practice moves towards shorter and more intensive radiotherapy treatment courses, online adaptive magnetic resonance-guided radiotherapy (MRgRT) has become an important technology to enable margin reductions and minimise major toxicities [1], [2]. In the evaluation of MRgRT tolerability, capturing changes in acute toxicity is important since acute and late moderate or worse toxicities may be associated [3]. Importantly, the methods used to assess acute toxicity influence the quality of the toxicity reports [1].

Despite advances in treatment technology, there is room for improvement in the current toxicity reporting in MRgRT, particularly from the patient perspective. Patient-reported outcomes (PROs) have emerged as an important supplementary assessment tool in both clinical trials and routine practice, providing insights into patient tolerability beyond clinician assessments [4], [5]. Incorporating multiple PROs has been suggested as a more comprehensive approach to evaluate tolerability, with the potential to improve the accuracy and clinical relevance of toxicity reporting in the context of online adaptive MRgRT [6].

Prostate cancer (PCa) has been one of the first tumour sites treated with MRgRT, making it an important setting to evaluate the use of PROs [7], [8], [9], complementing clinician reports of adverse events (Common Terminology Criteria for Adverse Events (CTCAE))[10]. A *meta*-analysis evaluated the added value of PROs in prostate MRgRT studies, including eleven observational studies and one randomised controlled trial (RCT) [11]. Importantly, none of these studies collected PROs during the four weeks following MRgRT; half of the studies assessed PROs at one-month follow-up [12], [13], [14], [15], [16], [17], while the others assessed them at 6–24 weeks after treatment [18], [19], [20], [21], [22], [23]. Later studies have continued to apply similar PRO assessment schedules [24], [25]. In four of the studies in the *meta*-analysis, low PRO response rates contributed to a critical risk of bias, and most trials enrolled patients with localised PCa exclusively [11]. One study included patients with low-volume metastatic (M1) disease, but PRO results were not reported separately for this subgroup [20]. Notably, for patients newly diagnosed with low-volume M1 PCa, MRgRT has become a treatment option, supported by evidence demonstrating improved survival with radiotherapy [26].

To fully understand the impact of MRgRT on patient health and health-related quality of life (HRQoL), comprehensive repeated assessments may provide us with more complete and nuanced toxicity profiles, moving beyond a single focus on the maximum grade of toxicity [27], [28], [29], [30]. Furthermore, real-time use of electronic PROs (ePROs) may increase completion rates and provide earlier identification of symptom deterioration, thus the possibility for more timely supportive care interventions [31].

Multiple factors may impair the HRQoL of PCa patients, including the level of acute symptoms and performance status [32], [33], [34], [35], [36], patient age (33) and use of androgen deprivation therapy (ADT) (36). Given the multifactorial influences on HRQoL, understanding how acute toxicities evolve over time and relate to HRQoL and patient characteristics is valuable.

Therefore, we hypothesised that more frequent ePRO assessments would improve the detection and characterization of prevalence, onset and persistence of acute toxicities during MRgRT and in the subsequent 12 weeks. The primary aim of this study was to prospectively evaluate acute symptom changes throughout MRgRT in patients with localized and low-volume M1 PCa, using weekly ePRO assessments integrated into the MRgRT workflow. Secondarily, we aimed to determine whether changes in HRQoL 12 weeks following MRgRT were associated with baseline clinical characteristics or increased symptom burden.

## Materials and methods

### Study design and participants

The PRO-MR-RT prospective observational cohort study was conducted at Odense University Hospital, Denmark, between November 2020 and December 2023. Eligible participants in this time period were adults (≥18 years) referred for MRgRT with either localised PCa or newly diagnosed low-volume M1 disease, cognitively able to provide informed consent and complete PROs in Danish. Patients with localized disease received moderate hypofractionation (60 Gy in 20 fractions, 5 fractions/week) per the PRISM trial [37]; low-volume M1 patients received 36 Gy in six fractions (2–3 fractions/week) based on Parker et al. [26]. Some patients in both cohorts received six months of ADT or longer initiated three months before radiotherapy. Treatment workflows followed established protocols (see Additional file 1). All participants received oral information about the study from the principal investigator (PKM) during their radiotherapy planning scans, and were provided with written study information. Written informed consent was subsequently obtained from all participants prior to study inclusion. The study was approved by the Danish Data Protection Agency (20/29991). The study was registered in ClinicalTrials.gov (NCT05615909).In addition, the participants were enrolled in the MOMENTUM study, a prospective international registry, where clinical and technical data are pseudonymised and stored [38].

### Data collection and ePRO measures

ePROs were collected using a validated pelvic symptom set with 18 symptomatic adverse events, scored on 4- or 5-point scales, captured digitally via the My Hospital platform and tested in a pilot study (Additional file 2) [39], [40]. Longitudinal repeated ePROs were collected at baseline, weekly during the 2–4 weeks of treatment, weekly for four weeks post-treatment, and at follow-up weeks four, eight, 12 and 24. HRQoL was assessed at baseline and at 12-week follow-up using the EuroQoL EQ-5D-5L and the EORTC QLQ-C30 questionnaires [41], [42] (Additional File 3. Fig. A1). HRQoL data 24 weeks after treatment were extracted from the Momentum Registry. Patients who were unable to complete digital entries were offered paper forms, which were manually entered by the study team. Baseline clinical and treatment characteristics were extracted from the electronic health records.

### PRO monitoring and clinical integration

My Hospital provided a secure, individualised patient interface, and PRO data were integrated into the electronic health record [43]. Radiation therapists reviewed graphical PRO summaries (green/yellow/red alerts for each symptom) weekly, the day after patient submission, to identify new or worsening symptoms and facilitate clinical follow-up (Additional file 2).

### Outcomes

The primary outcome was the longitudinal increase or decrease in patient-reported symptoms. Secondary outcomes were the proportion of patients having moderate to very severe urinary and bowel symptoms (scores 2–4 on PRO-CTCAE, 3–4 on EORTC) over time. Secondary outcomes also included changes in HRQoL metrics (EQ-5D index score, EQ-VAS, EORTC QLQ-C30 scales) and association of EQ-VAS with clinical parameters (age, ADT use or treatment fractionation) or symptom worsening. Clinician compliance rates reviewing ePROs in the clinic, and patient response rates were extracted from My Hospital software.

### Statistical analysis

Analyses were stratified by treatment cohort. Descriptive statistics summarised baseline characteristics and symptom prevalence. Linear mixed-effects models with patient ID as a random effect and time as a fixed effect estimated longitudinal symptom trajectories, accounting for missing data. Change scores of HRQoL followed established scoring manuals [44], [45]. The clinical relevance of changes in HRQoL results was interpreted based on the minimally important difference (MID) estimate of 0.08 for EQ-5D index score and MID of 7 points for EQ VAS scores reported by Pickard et al. [46]. The EORTC QLQ-C30 scales were interpreted based on MIDs from Cocks et al.[47]. The proportion of patients having symptom changes was calculated based on the number of patients who provided valid PRO assessments at each designated time point according to Setting International Standards in Analysing Patient-Reported Outcomes and Quality of Life Endpoints (SISAQOL) recommendations [48]. For the HRQoL outcomes, between-group comparisons of continuous outcomes were performed using Welch’s two-sample *t*-test. Between-group comparisons of non-parametric data were analysed using the Wilcoxon rank-sum test (Mann–Whitney U). Within-group changes over time were assessed using the Wilcoxon signed-rank test for paired data.

We modelled change in EQ-VAS from baseline to week 12 (ΔEQ-VAS) using separate linear regressions for each exposure. The pre-specified adjustment set was baseline EQ-VAS and performance status (PS; 1–2 vs 0). Exposures were symptom-worsening indicators from PRO-CTCAE/EORTC (mild (1-point) or moderate-severe (>1-point) worsening vs no worsening) and clinical covariates (age > 70, 36 Gy/6fx vs 60 Gy/20fx, concomitant ADT vs none). Only patients completing both baseline and week-12 EQ-VAS were included in the analysis. Results are presented as mean differences in ΔEQ-VAS with 95 % confidence intervals (CIs) in a forest plot.

Exploratory analyses followed SISAQOL recommendations [39], [48]. No formal power calculation was performed due to the exploratory design. Analyses were conducted in R (version 2023.09.0) [49].

## Results

Of 94 eligible patients informed about the study, three (mean age 81) declined participation as they did not feel capable of managing the questionnaire burden. Of the 91 who accepted, four dropped out due to non-compliance, seven were excluded for disease or treatment-related reasons, and four with low-volume PCa were excluded due to early-phase treatment differences (see Additional File 3, Fig. A.2).

The final sample consisted of 76 patients (mean age, 70), with 45 % (n = 34) having low-volume M1 disease. Most (81 %) had a WHO Performance Status of 0, and 66 % received concomitant ADT (Table 1). The ePRO response rates during treatment were high (94–100 % in the 36 Gy cohort, 93–100 % in the 60 Gy cohort). The majority of patients (76,5%) responded to 80 % or more of questionnaires in the 36 Gy Group and 75 % of questionnaires (76,2% of patients) in the 60 Gy group (Additional file 3. Fig. A.3). The 6-month retention rate was 82 % and 89 %. Four patients (5 %) declined ePROs and completed paper-based questionnaires. Radiation therapists had a 97 % compliance rate in reviewing ePROs and initiating weekly supportive care when needed.

**Table 1:** Baseline characteristics of patients with PCa treated with MRgRT (n = 76).

Characteristics, n (%)	Total Pca group		Low-volume M1 Pca 36 Gy/6 Fx		Localised Pca 60 Gy/20 Fx	
n=	76	100 %	34	45 %	42	55 %
Age, mean (range)	69.6	(56–81)	70	(60–81)	69	(56–76)
*WHO, performance status*						
0	62	81 %	29	85 %	33	79 %
1	12	16 %	4	12 %	8	19 %
2	2	3 %	1	3 %	1	2 %
*Pre-treatment PSA, median (IQR)*						
before radiotherapy	8.8 (13.2)		14 (15.6)		6.0 (8.9)	
before ADT	21 (47.7)		49 (82.0)		8.1 (11)	
*Gleason score*						
6	1	1 %	1	3 %	0	0 %
7	57	75 %	15	44 %	42	100 %
8	5	7 %	5	15 %	0	0 %
9	13	17 %	13	38 %	0	0 %
*T-stage*						
T1	28	37 %	2	6 %	26	62 %
T2	18	24 %	4	12 %	14	33 %
T3	24	31 %	24	70 %	0	0 %
T4	3	4 %	3	9 %	0	0 %
Not applicable	3	4 %	1	3 %	2	5 %
*N-stage*						
N0	58	76 %	16	47 %	42	100 %
N1	13	17 %	13	38 %	0	0 %
Nx	5	7 %	5	15 %	0	0 %
*M−stage*						
M0	42	55 %	0	0 %	42	100 %
M1	34	45 %	34	100 %	0	0 %
*Conc. systemic treatment*						
None	26	34 %	1	3 %	25	60 %
≤ 6 months	16	21 %	0	0 %	16	38 %
> 6 months	34	45 %	33	97 %	1	2 %

### Urinary symptom changes during and after MRgRT

**Fig. 1:**
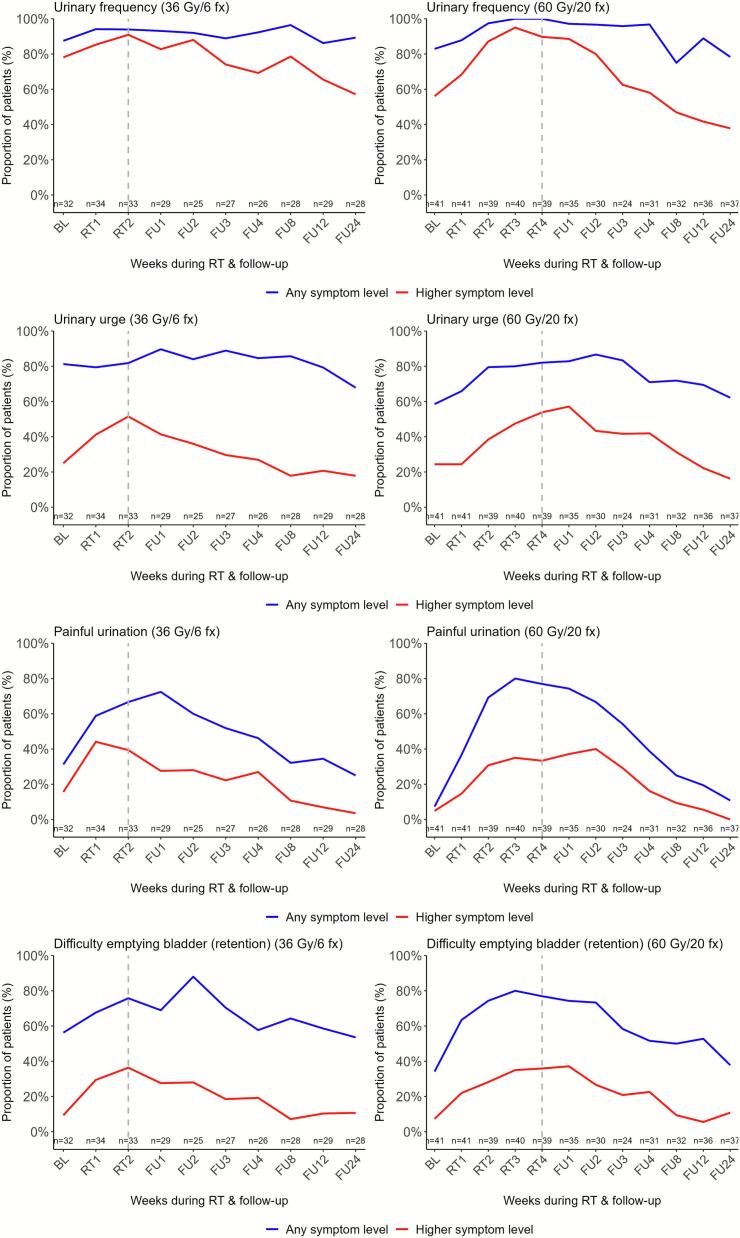
**Longitudinal proportions of patient-reported urinary toxicity significantly changing during or after MRgRT (n = 76)**. "Any symptom level" includes scores of ≥1 on PRO-CTCAE and ≥2 on EORTC QLQ-C30 scales. “Higher symptom level” includes scores of ≥2 on PRO-CTCAE (‘moderate’ to ‘very severe’ or ‘occasionally’ to ‘almost constantly’) and ≥3 on EORTC QLQ-C30 (‘quite a bit’ to ‘very much’) scales. The dotted vertical line marks the last week of radiotherapy. Abbreviations: BL, baseline; RT, radiotherapy; FU, follow-up.

Before MRgRT, urinary frequency was the most common symptom, reported occasionally or more often by 74 % (36 Gy cohort) and 56 % (60 Gy cohort). During treatment, reports of moderate-to-severe urge, painful urination, and retention more than doubled in both cohorts but largely resolved by week 8 (36 Gy) and week 12 (60 Gy) (Fig. 1. Additional File 3, Table A.1).

Linear mixed models showed significant worsening from baseline in all symptoms but one (urinary incontinence) urinary symptom. In the 36 Gy cohort, mainly painful urination emerged in treatment week 1 (p < 0.001) and persisted to follow-up week 1 (p = 0.002); urinary retention persisted until follow-up week 2 (p = 0.013) (Fig. 1. Additional File 3, Table A.2). In the 60 Gy cohort, retention was the first significant urinary symptom changing (week 1, p = 0.028), followed by urinary frequency, urge, and painful urination from weeks 2–3, persisting through follow-up weeks 3–4 (e.g., urge, p = 0.019, painful urination, p = 0.001) (Fig. 1. Additional File 3, Table A.2).

### Bowel symptom changes during and after MRgRT

**Fig. 2:**
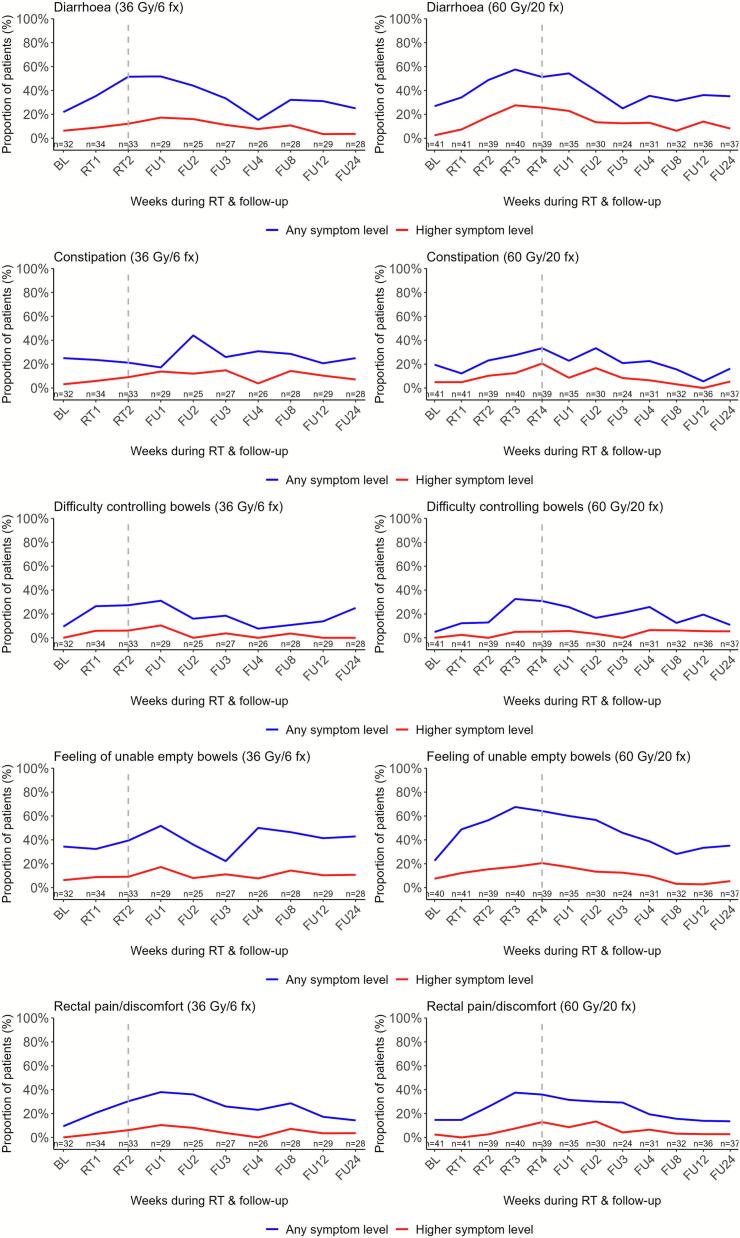
**Longitudinal proportions of patient-reported bowel toxicity significantly changing during or after MRgRT (n = 76)**. "Any symptom level" includes scores of ≥1 on PRO-CTCAE and ≥2 on EORTC QLQ-C30 scales. “Higher symptom level” includes scores of ≥2 on PRO-CTCAE (moderate to very severe or occasionally to almost constantly) and ≥3 on EORTC QLQ-C30 (quite a bit to very much) scales. The dotted vertical line marks the last week of radiotherapy. Abbreviations: BL, baseline; RT,radiotherapy; FU, follow-up.

Before treatment, a maximum of 8 % reported moderate or worse bowel symptoms except for a decreased appetite in the 36 Gy cohort (13 %). Diarrhoea became the most prevalent bowel toxicity during MRgRT, reported by 17 % (36 Gy cohort) and 28 % (60 Gy cohort) of responders as a frequent symptom (Fig. 2). A notable proportion of patients reported a sensation of incomplete bowel emptying — 17 % and 21 % in the respective cohorts — as well as constipation (21 %) in the 60 Gy cohort, particularly at the end of treatment or in the weeks following (Fig. 2. Additional File 3, Table A.1).

Linear mixed model analyses indicated significantly increased bowel symptoms, especially during follow-up: in the 36 Gy cohort where diarrhoea (p = 0.029) and difficulty controlling bowels (p = 0.004) peaked in follow-up week 1 and constipation (p = 0.002) peaked in week 2. In the 60 Gy cohort, diarrhoea (p < 0.001), nausea (p = 0.035), and difficulty controlling bowels (p = 0.004) worsened from week 3, with diarrhoea persisting into follow-up week one (p = 0.014) (Fig. 2. Additional File 3, Table A.2. This cohort also reported a significantly increased feeling of incomplete bowel emptying from treatment week 2 (p = 0.011), persisting to follow-up week 1 (p = 0.008).

### Quality of life outcomes after MRgRT

HRQoL questionnaires were completed at baseline and follow-up by 74 % of the patients (n = 56). At baseline, HRQoL did not significantly differ between the two treatment cohorts (EQ-5D index score, p = 0.327; EQ-VAS, p = 0.209) (Table 2). However, at the 12- and 24-week follow-up, the calculated EQ-5D index score significantly differed between the two cohorts (p = 0.049, p = 0.016). There was a clinically relevant improvement in the median EQ-5D index score for patients with localised disease at week 12 and 24 (+0.08 + 0.14) but no clinically relevant change in the 36 Gy cohort. A small decrease for this group was largely due to 23 % of low-volume M1 PCa patients reporting worsening in most EQ-5D dimensions at both follow-ups (Additional File 3, Table A.3). Interestingly, for this cohort, a small but non-significant improvement was seen in the patients’ self-rated health week 24 (EQ-VAS, +4.5; p = 0.606). Also, a small clinically relevant improvement was detected in week 12 in their self-rated QLQ-C30 global health state/QoL (+6.0) and emotional functioning(+6.1). Emotional functioning improved in the 60 Gy group at week 24 (+7.8). Insomnia was the only symptom score being reduced beyond thresholds from baseline to week 12 in both groups (–8.1, –6.4 points) (Table 2).

**Table 2:** Health-related quality of life outcomes of patients with PCa treated with MRgRT (n = 76).

**Respondents, n(%)**	36 Gy/6 fx (n = 34)	60 Gy/20 fx (n = 42)	*p-value*
Baseline	39 (93 %)	29 (85 %)	
Week 12	36 (86 %)	29 (85 %)	
Week 24	21 (62 %)	26 (62 %)	
**Outcomes**			
**EQ5D index score** (score range 0–1, 1 = highest HRQoL), median (IQR)			
Baseline, mean (SD)	0.836 (0.12)	0.872 (0.09)	
Week 12	0.825 (0.16)	0.896 (0.12)	
Week 24	0.821 (0.13)	0.906 (0.12)	
Baseline, median (IQR)	0.829 (0.13)	0.859 (0.20)	*0.327^c^*
Week 12	0.798 (0.27)	0.935 (0.18)	*0.049^c^*
Week 24	0.787 (0.15)	1.000 (0.21)	*0.016^c^*
*Within-group difference in median score^a^*	*p = 0.349*	*p = 0.216*	
**EQ5D-VAS** (score range 0–100, 0 = worst posible health, 100 = best possible health), mean (SD)			
Baseline	76.5 (14.2)	81.2 (15.0)	*0.209^b^*
Week 12	76.1 (14.0)	81.6 (13.2)	*0.119^b^*
Week 24	81.0 (12.7)	81.8 (16.0)	*0.825^b^*
*Within-group difference in mean score 0*–*24 weeks^b^*	*p = 0.606 (n=20)*	*p = 0.442 (n=27)*	
**EORTC QLQ-C30 summary score** (range 0–100, high 0 better), mean (SD)			
Baseline	87.4 (8.5)	90.4 (7.6)	*0.145*
Week 12	87.3 (10.7)	91.1 (8.6)	*0.123*
Week 24	86.9 (7.7)	91.6 (9.6)	*0.063*
**EORTC QLQ-C30 Global Health status**			
Baseline	66.1 (18.4)	75.2 (18.8)	
Week 12	72.1 (15.8)	77.3 (15.9)	
Week 24	70.8 (16.8)	76.9 (20.0)	
**EORTC QLQ-C30 Physical functioning**			
Baseline	88.5 (16.6)	92.3 (9.4)	
Week 12	85.3 (18.7)	92.4 (10.5)	
Week 24	88.1 (13.3)	88.5 (14.8)	
**EORTC QLQ-C30 Emotional functioning**			
Baseline	80.7 (16.4)	87.2 (14.8)	
Week 12	86.8 (13.5)	92.1 (10.5)	
Week 24	86.5 (12)	95.0 (8.9)	
**EORTC QLQ-C30 Role functioning**			
Baseline	86.8 (22.9)	91.9 (17.0)	
Week 12	84.5 (23.1)	89.8 (19.6)	
Week 24	84.0 (21.1)	90.6 (20.4)	
**EORTC QLQ-C30 Social functioning**			
Baseline	90.8 (15.2)	92.7 (13.1)	
Week 12	89.7 (17.5)	93.5 (10.7)	
Week 24	86.2 (13.9)	93.9 (12.7)	
**EORTC QLQ-C30 Cognitive functioning**			
Baseline	88.5 (16.1)	91.0 (13.7)	
Week 12	88.5 (14.8)	91.7 (17.1)	
Week 24	88.2 (11.5)	87.8 (19.0)	
**Symptom scales** (high score for a symptom scale / item represents a high level of symptomatology/problems)			
**Insomnia**			
Baseline	32.2 (30.2)	23.1 (27.7)	
Week 12	24.1 (26.6)	16.7 (21.8)	
Week 24	29.2 (24.7)	15.6 (22.7)	
*^a^Wilcoxon signed-rank test*			
*^b^t-test/paired t-test*			
*^c^Mann-Whitney U test*			

**Fig. 3:**
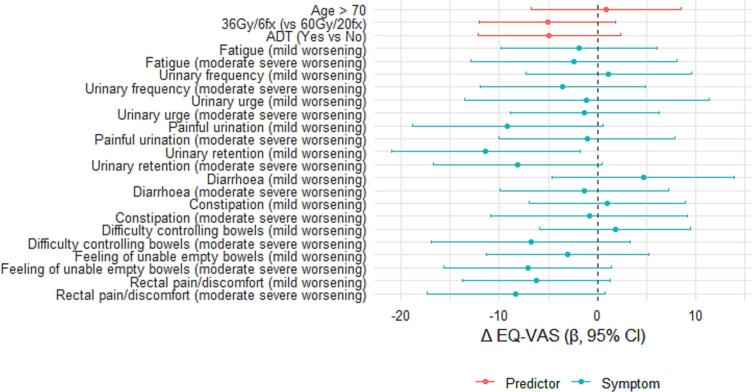
**Adjusted associations with changes in self-rated health (EQ-VAS) from baseline to week 12 after MRgRT (n = 56)**. Points show the mean difference in ΔEQ-VAS (week 12 minus baseline) from separate linear models for each exposure with 95% adjusted for baseline EQ-VAS and PS. Mild symptom worsening = 1-point increase (to max 2 in the EORTC items). Moderate or severe symptom worsening = 2-point increase or higher (or 1-point to 3 or 4 in EORTC items).

In the linear regression analysis of changes in EQ-VAS (ΔEQ-VAS), patients having had symptom worsening generally showed lower ΔEQ-VAS (less improvement) than those without worsening after adjusting each exposure for baseline EQ-VAS and performance status (PS 1–2 vs 0) (Fig. 3). However, most of the 95 % CIs included zero. Clinical predictors (age > 70, low vs high radiation dose, concomitant ADT) showed little evidence of association after adjustment. Only for those reporting mild worsening in urinary retention, the association with decreased EQ-VAS was significant (p = 0.020). Overall, effect sizes were modest and estimates imprecise, consistent with the sample size (Fig. 3).

## Discussion

This study uniquely reports on multiple ePRO assessments during the acute phase of MRgRT for both localised and low-volume M1 PCa patients, offering comprehensive insights into symptom trajectories. Multiple assessments revealed that patients with low-volume M1 PCa undergoing ultra-hypofractionated MRgRT experienced peak urinary symptoms at treatment completion and two weeks thereafter, while bowel symptoms peaked in the first and second week of follow-up. Conversely, patients with localised disease receiving moderate hypofractionation reported peak urinary and bowel symptoms in the third week of treatment, with significant changes in urinary symptoms persisting up to four weeks after treatment.

Our findings underscore the importance of integrating frequent, patient-reported assessments during and immediately after MRgRT to accurately capture the trajectory of acute toxicity. Previous studies, like data from the MOMENTUM dataset collected its first post-baseline clinician and patient-reported assessments only at three months, well after the expected acute toxicity peak around weeks two to five, as suggested by larger trials such as CHHiP and PACE-B [27], [50], [51]. This likely resulted in undetected maximal acute toxicity events and potential recall bias, where some patients reported their worst symptoms retrospectively while others described only current symptoms at the time of assessment. Similar time gaps between assessments were highlighted in the *meta*-analysis of PROs in prostate MRgRT studies. Maximum patient-reported toxicity was reported at the end of treatment or one month following ultra-hypofractionated prostate MRgRT since no PRO assessments were collected between these time points [11]. All these infrequent PRO assessments have led to papers recommending patients should be informed about an expected peak in acute toxicity 1 month following MR-guided SBRT which is not coherent with the findings in this study [25].

Our study’s approach of frequent, repeated PRO measurements provides a more nuanced picture of symptom development instead of risking recall bias and undetected symptom deterioration between in-person visits enabling more precise toxicity monitoring, patient-centered care adjustments, and ultimately a deeper understanding of treatment tolerability.

The observed small improvement in self-reported QoL among patients with low-volume M1 PCa contrasts with findings from the STAMPEDE trial, where QoL declined [26]. Notably, our study's shorter radiotherapy courses (2–3 weeks) as well as not including patients with high volume metastatic burden may contribute to this difference. However, similar improvement in emotional functioning was found six and 12 weeks after ultrahypofractionated MRgRT for a cohort of patients with localised PCa [24]. Although the improvements in our study was small, they suggest a trend toward improved emotional well-being for this cohort during follow-up. How the acute toxicity correlates with HRQoL cannot be concluded with this sample size, however, the findings could indicate that having had acute worsening of symptoms in the months following MRgRT does not affect the QoL of patients at the 12-week follow-up.

A major strength of our study is the high patient adherence to ePROs, likely facilitated by real-time monitoring and feedback from radiation therapists. This reinforces the value of patient and staff engagement in the development and integration of ePROs as our previous pilot study confirmed a patient need for active feedback for PROs to make sense [4], [40]. The development of a concise, treatment-specific item set tailored for PCa patients undergoing MRgRT further enhanced the relevance and manageability of assessments. Studies relying solely on PRO-CTCAE may overlook important symptoms such as urinary retention, frequency, and nocturia, as these are not included in the PRO-CTCAE item library—highlighting the limitation that patient-reported outcomes can only capture the issues we explicitly ask about [25].

The patient compliance in our study was high (79–100 %). Previous studies in the *meta*-analysis investigating PROs in the prostate MRgRT workflow found missing patient-reports to be serious or critical in one third of the studies with completion rates varying from 17-100 % [11]. This is critical if PROs are to be used to measure the impact of toxicity-minimizing treatments in MRgRT clinical trials [5]. None of the studies used the patient-reports actively which is known to reduce questionnaire completion rates [4] and must be taken into account in future trials.

However, the small sample size and the absence of a control group limits causal inferences, and reliance on digital reporting may introduce selection bias which was not the case in this study. Only prostate patients were included limiting the generalizability to other treatment groups. A limitation of the study is that only white male patients were included, reflecting the demographic of those referred for this specific treatment during the study period. Future studies should aim to include control groups and explore methods to ensure broader patient inclusion. Future research should prioritise the integration of high-quality PROs alongside traditional clinical data to enhance treatment evaluation and decision-making. Also, research should focus on investigating PRO-driven algorithms with active feedback mechanisms that are effective in guiding timely supportive care interventions to minimise the severity of acute toxicities [52], [53].

## Conclusion

This study delivers the first detailed characterization of acute symptom trajectories during and after MRgRT in localised and low-volume metastatic PCa, captured through frequent ePRO assessments. The prevalence, timing, and persistence of urinary and bowel toxicities varied by treatment schedule. Several symptoms peaked in the immediate weeks post-radiotherapy, underscoring the need for close monitoring within this timeframe. Importantly, moderate symptom worsening did not translate into decreased HRQoL. As ultra-hypofractionation expands, these findings highlight the critical role of symptom-guided, patient-centered MRgRT workflows and personalised remote care strategies.

## Funding sources

This study was funded by the Novo Nordisk Foundation (NNF18OC0052974) and AgeCare (Academy of Geriatric Cancer Research). However, the sources of funding did not influence the design of the study, writing of the manuscript, nor the collection, analysis and interpretation of the data.

## Declaration of competing interest

The authors declare that they have no known competing financial interests or personal relationships that could have appeared to influence the work reported in this paper.
