# Supplementary material for: Optimising acute toxicity monitoring in prostate MR-guided radiotherapy workflow: Results from a prospective study using multiple electronic PRO assessments
Source: Tech Innov Patient Support Radiat Oncol. 2025 Dec 10;37:100368. doi: 10.1016/j.tipsro.2025.100368 (PMC12774778; doi:10.1016/j.tipsro.2025.100368)
Supplement: Supplementary Data 1 [file mmc1.docx]

## Additional File 1. Treatment guidelines MR-linac

**The MR-linac workflow**

All patients were treated with an adapt-to-shape workflow. During each treatment fraction, a T2w 3D MR scan was acquired, and contours were propagated from the pre-treatment MR to the daily session MR using deformable image registration. Manual corrections of the transferred contours were usually needed before treatment plan adaptation optimisation was initiated. A second MR scan was acquired during plan adaptation for position verification. If the target had moved more than 2 mm, a second plan adaptation process was initiated, and a new position verification scan was acquired.

All patients were instructed to empty their bladder one hour before radiotherapy and drink 3-500 ml of water.

## CTV to PTV margins, OAR constraints, and target coverage requirements for patients with localised PCa

The primary clinical target volume (CTV) was defined as the prostate and proximal 1 cm of the seminal vesicles.

The secondary CTV comprised the proximal 2 cm of the seminal vesicles exterior to the primary CTV.

| PTV margin | Prior to January 2022:  PTV1 = CTV1 + 5 mm left, right, superior, inferior, ant, and 3 mm post PTV2 = CTV2 + 0,5 cm isotropic  From January 2022:  PTV1 = CTV1 + 3 mm left, right, 4 mm superior, inferior, and 5 mm ant and post PTV2 = CTV2 + 3 mm left, right, 4 mm superior, inferior, and 5 mm ant and post |
| --- | --- |
| Target coverage requirements | CTV1(60Gy): V95%=100%, mean dose=99%-101%  PTV1(60Gy): V95%>99%, V90%=100%, V107%=0%  CTV2(48,6Gy): V95%=100%  PTV2(48,6Gy): V95%>99%, V90%=100%  External-PTV1 V105%=0% |
| OAR dose constraints | \| Organ at risk \| Dose (Gy) \| Max Volume (% or cc) \| \| \| --- \| --- \| --- \| --- \| \|  \|  \| Optimal \| Mandatory \| \| Rectum \| 24.4  32.4  40.5  47.0  48.6  52.7  56.8  60.8  61.8 \| 80%  65%  50%    35%  -    3% \| -  -  60%  *  50%  30%  15%  5%  0% \| \| Bladder \| 40.5  48.7  52.7  56.76  60.8 \| 50%  25 %    5%  3% \| 50%  35%  25% \| \| Femoral head \| 40.5 \|  \| 50% \| \| Bowel \| 36.5  40.5  44.6  48.7  52.7 \| 78cc  17cc  14cc  0.5cc \| 158cc  110cc  28cc  6cc  <0.01cc \| \| Penile Bulb \| 40.5 \|  \| 50% \|   *47 Gy isodose line may not surround the circumference of the rectum in any horizontal slice. |
| Prioritise | 1) OAR mandatory 2) Coverage of PTV 3) Remaining OAR |

## CTV to PTV margins, OAR constraints, and target coverage requirements for patients with low-volume metastatic disease

The CTV for the patients treated with 36 Gy/6 Fx consisted of the prostate and the visible extra-prostatic tumour tissue.

| PTV margin | Prior to February 2022:  PTV = CTV + 5 mm left, right, 4 mm superior, inferior, and 5 mm ant and post  From February 2022:  PTV = CTV + 3 mm left, right, 4 mm superior, inferior, and 5 mm ant and post |
| --- | --- |
| Target coverage requirements | CTV(36Gy): V95%=100%, mean dose=99%-108%  PTV(36Gy): V95%>98%, V120%<0.01cc |
| OAR dose constraints | \| Organ at risk \| Dose (Gy) \| Max Volume (% or cc) \| \| \| --- \| --- \| --- \| --- \| \|  \|  \| Optimal \| Mandatory \| \| Rectum \| 36  34.2  32.4  25  20 \| 0.1cc  3cc  7cc  25cc \| 0.3cc  6cc  10cc  30cc  * \| \| Bladder \| 36  35  33 \| 0.3cc  5cc  20cc \| 0.6cc  7.5cc  25cc \| \| PRV_Urethra (Urethra+3mm) \| 36 \| 0.3cc \| 0.6cc \| \| Femoral head \| 20 \| 1% \| 2% \| \| Bowel \| 34.4  32.4  20 \| 0.1cc  1cc  5cc \| 0.5cc  3cc  15cc \| \| Penile Bulb \| 25 \|  \| 50% \|   *20 Gy isodose line may not surround the circumference of the rectum in any horizontal slice. |
| Prioritise | 1. PRV Urethra 2. Rectum 3. CTV coverage 4. Remaining OAR 5. PTV coverage |
