# Supplementary material for: Optimising acute toxicity monitoring in prostate MR-guided radiotherapy workflow: Results from a prospective study using multiple electronic PRO assessments
Source: Tech Innov Patient Support Radiat Oncol. 2025 Dec 10;37:100368. doi: 10.1016/j.tipsro.2025.100368 (PMC12774778; doi:10.1016/j.tipsro.2025.100368)
Supplement: Supplementary Data 2 [file mmc2.docx]

**Additional file 2.** The PROM and the ePRO graphical summary from My Hospital software

| The PRO pelvic item set developed and validated for prostate MRgRT^1^ | | | | | | |
| --- | --- | --- | --- | --- | --- | --- |
| Worst symptom | Which symptom affects your daily life the most right now? | Free-text write-in | | | | |
| *PRO-CTCAE*  *symptomatic AE* | *PRO-CTCAE question* | *Response option (score 0-4)* | | | | |
|  |  | **0** | **1** | **2** | **3** | **4** |
| Decreased appetite | In the last 7 days, what was the SEVERITY of your DECREASED APPETITE at its WORST? | None | Mild | Moderate | Severe | Very Severe |
|  | In the last 7 days, how much did DECREASED APPETITE INTERFERE with your usual or daily activities? | Not at all | A little bit | Somewhat | Quite a bit | Very Much |
| Nausea | In the last 7 days, how OFTEN did you have NAUSEA? | Never | Rarely | Occasionally | Frequently | Almost constantly |
|  | In the last 7 days, what was the SEVERITY of your NAUSEA at its WORST? | None | Mild | Moderate | Severe | Very Severe |
| Constipation | In the last 7 days, what was the severity of your CONSTIPATION at its WORST? | None | Mild | Moderate | Severe | Very Severe |
| Diarrhoea | In the last 7 days, how often did you have LOOSE OR WATERY STOOLS (DIARRHOEA)? | Never | Rarely | Occasionally | Frequently | Almost constantly |
| Abdominal pain | In the last 7 days, how often did you have PAIN IN THE ABDOMEN (BELLY AREA)? | Never | Rarely | Occasionally | Frequently | Almost constantly |
|  | In the last 7 days, what was the severity of your PAIN IN THE ABDOMEN (BELLY AREA) at its WORST? | None | Mild | Moderate | Severe | Very Severe |
|  | In the last 7 days, how much did PAIN IN THE ABDOMEN (BELLY AREA) INTERFERE with your usual or daily activities? | Not at all | A little bit | Somewhat | Quite a bit | Very Much |
| Radiation skin reaction | In the last 7 days, what was the SEVERITY of your SKIN BURNS FROM RADIATION at their WORST? | None | Mild | Moderate | Severe | Very Severe |
| Fatigue | In the last 7 days, what was the SEVERITY of your FATIGUE, TIREDNESS, OR LACK OF ENERGY at its WORST? | None | Mild | Moderate | Severe | Very Severe |
|  | In the last 7 days, how much did FATIGUE, TIREDNESS, OR LACK OF ENERGY INTERFERE with your usual or daily activities? | Not at all | A little bit | Somewhat | Quite a bit | Very Much |
| Painful urination | In the last 7 days, what was the severity of YOUR PAIN OR BURNING WITH URINATION at its WORST? | None | Mild | Moderate | Severe | Very Severe |
| Urinary frequency | In the last 7 days, were there times when you had to urinate frequently? | Never | Rarely | Occasionally | Frequently | Almost constantly |
|  | In the last 7 days, how much did FREQUENT URINATION INTERFERE with your usual or daily activities? | Not at all | A little bit | Somewhat | Quite a bit | Very Much |

| EORTC symptomatic AE | EORTC question | Response option (score 1-4) | | | | | | | | | | | | | | |
| --- | --- | --- | --- | --- | --- | --- | --- | --- | --- | --- | --- | --- | --- | --- | --- | --- |
| Pain/discomfort around anal opening  (rectal pain/discomfort) | During the past week: Have you had pain/discomfort around your anal opening (back passage)? | Not at all | | | A little | | | | Quite a bit | | | | Very Much | | | |
| Frequent urination at night (nocturia) | During the past week: Have you had to urinate frequently at night? | Not at all | | | A little | | | | Quite a bit | | | | Very Much | | | |
| Unintentional release (leakage) of urine (urinary incontinence) | During the past week: Have you had any unintentional release (leakage) of urine? | Not at all | | | A little | | | | Quite a bit | | | | Very Much | | | |
| Difficulty emptying bladder (retention) | During the past week: Have you had difficulty emptying your bladder? | Not at all | | | A little | | | | Quite a bit | | | | Very Much | | | |
| Urinary urge | During the past week: When you felt the urge to pass urine, did you have to hurry to get to the toilet? | Not at all | | | A little | | | | Quite a bit | | | | Very Much | | | |
| Bloated feeling in abdomen | During the past week: Have you had a bloated feeling in your abdomen? | Not at all | | | A little | | | | Quite a bit | | | | Very Much | | | |
| Difficulty controlling bowels | During the past week: Have you had difficulty in controlling your bowels? | Not at all | | | A little | | | | Quite a bit | | | | Very Much | | | |
| Blood in stools | During the past week: Have you had blood in your stools? | Not at all | | | A little | | | | Quite a bit | | | | Very Much | | | |
| Unable empty bowels | During the past week: Have you had the feeling of being unable to completely empty your bowels? | Not at all | | | A little | | | | Quite a bit | | | | Very Much | | | |
| **ADDITIONAL ITEMS measured at baseline, 4, 8, 12 and 24 weeks following radiotherapy** | | | | | | | | | | | | | | | | |
| PRO-CTCAE  symptomatic AE | PRO-CTCAE question | Response option (score 0-6) | | | | | | | | | | | | | |  |
| Achieve and maintain erection | In the last 7 days, what was the severity of your DIFFICULTY GETTING OR KEEPING AN ERECTION at its WORST? | None | Mild | | | Moderate | | Severe | | Very Severe | | Not sexually active | | | Prefer not to answer |  |
| Decreased libido | In the last 7 days, what was the SEVERITY of your DECREASED SEXUAL INTEREST at its WORST? | None | Mild | | | Moderate | | Severe | | Very Severe | | Not sexually active | | | Prefer not to answer |  |
| **OTHER SYMPTOMS** | | | | | | | | | | | | | | | |  |
| PRO-CTCAE | PRO-CTCAE Question |  | | | | | | | | | | | | | |  |
| Other symptom | Do you have any other symptoms that you wish to report? | Yes/No | | | | | | | | | | | | | |  |
|  | If ‘Yes’: Please list any other symptoms | Free-text write-in | | | | | | | | | | | | | |  |
|  | If ‘Yes’: | Response option (score 0-4) | | | | | | | | | | | | | |  |
| Other symptom 1 | In the last 7 days, what was the SEVERITY of this symptom at its WORST? | None | | Mild | | | Moderate | | | | Severe | | | Very severe | |  |
| Other symptom 2 | In the last 7 days, what was the SEVERITY of this symptom at its WORST? | None | | Mild | | | Moderate | | | | Severe | | | Very severe | |  |
| Other symptom 3 | In the last 7 days, what was the SEVERITY of this symptom at its WORST? | None | | Mild | | | Moderate | | | | Severe | | | Very severe | |  |
| Other symptom 4 | In the last 7 days, what was the SEVERITY of this symptom at its WORST? | None | | Mild | | | Moderate | | | | Severe | | | Very severe | |  |
| Other symptom 5 | In the last 7 days, what was the SEVERITY of this symptom at its WORST? | None | | Mild | | | Moderate | | | | Severe | | | Very severe | |  |

^1^Møller PK, Pappot H, Bernchou U, Schytte T, Dieperink KB. Development of patient-reported outcomes item set to evaluate acute treatment toxicity to pelvic online magnetic resonance-guided radiotherapy. Journal of Patient-Reported Outcomes. 2021;5(1):47


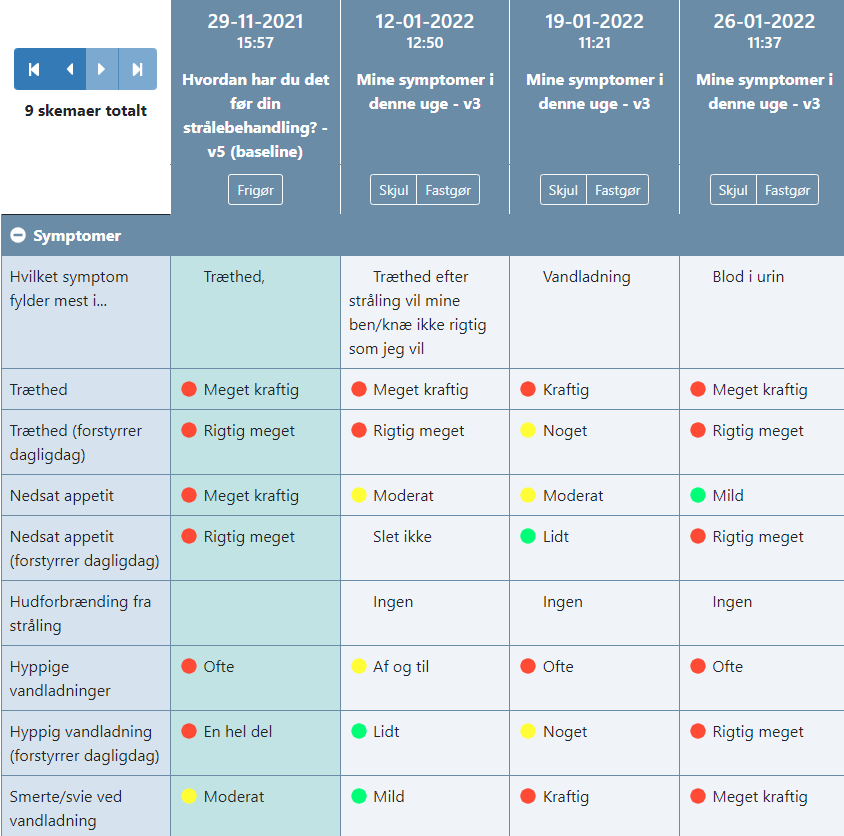
Example from My Hospital software (graphical summary data translated to English)

None

None

None

Very severe

Severe

Moderate

Mild

Blood in urine

Urination

Fatigue – after the treatment

Fatigue

**Baseline**

**RT week 1**

**RT week 2**

**RT week 3**

A little bit

Very much

Frequently

Quite a bit

Mild

Frequently

Frequently

Very much

Very much

Very much

Not at all

Very much

Very much

A little bit

Occasionally

Moderate

Somewhat

Somewhat

Severe

Very severe

Very severe

Moderate

Very severe

Very severe

Painful urination

Frequent urination (interference)

Frequent urination

Radiation skin reaction

Decreased appetite (interference)

Which symptom affects your daily life the most right now?

Fatigue (severity)

Fatigue (interference)

Decreased appetite

Symptoms
