# Supplementary material for: Optimising acute toxicity monitoring in prostate MR-guided radiotherapy workflow: Results from a prospective study using multiple electronic PRO assessments
Source: Tech Innov Patient Support Radiat Oncol. 2025 Dec 10;37:100368. doi: 10.1016/j.tipsro.2025.100368 (PMC12774778; doi:10.1016/j.tipsro.2025.100368)
Supplement: Supplementary Data 3 [file mmc3.docx]

**Additional File 3. Supplementary figures and tables**

**Figure A.1. Data collection in the PRO-MR-RT study**

**
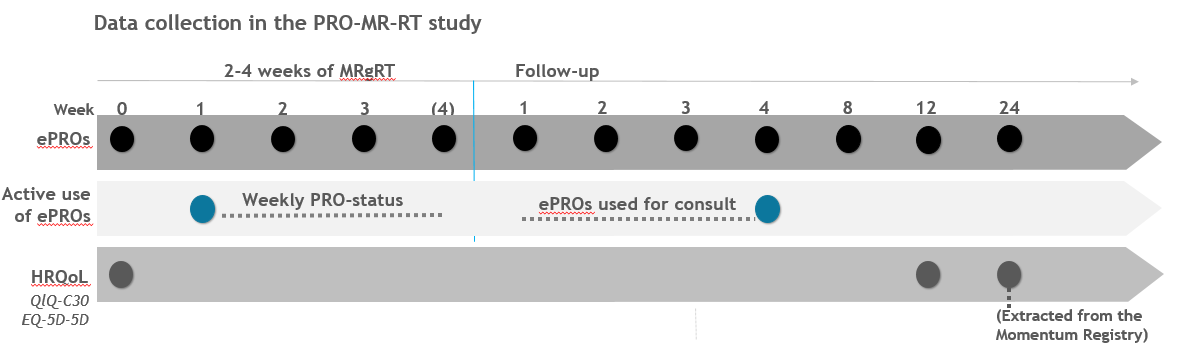
**

**Figure A.2.** Flowchart of PCa patients treated with online adaptive MRgRT in the PRO-MR-RT study (n=76)


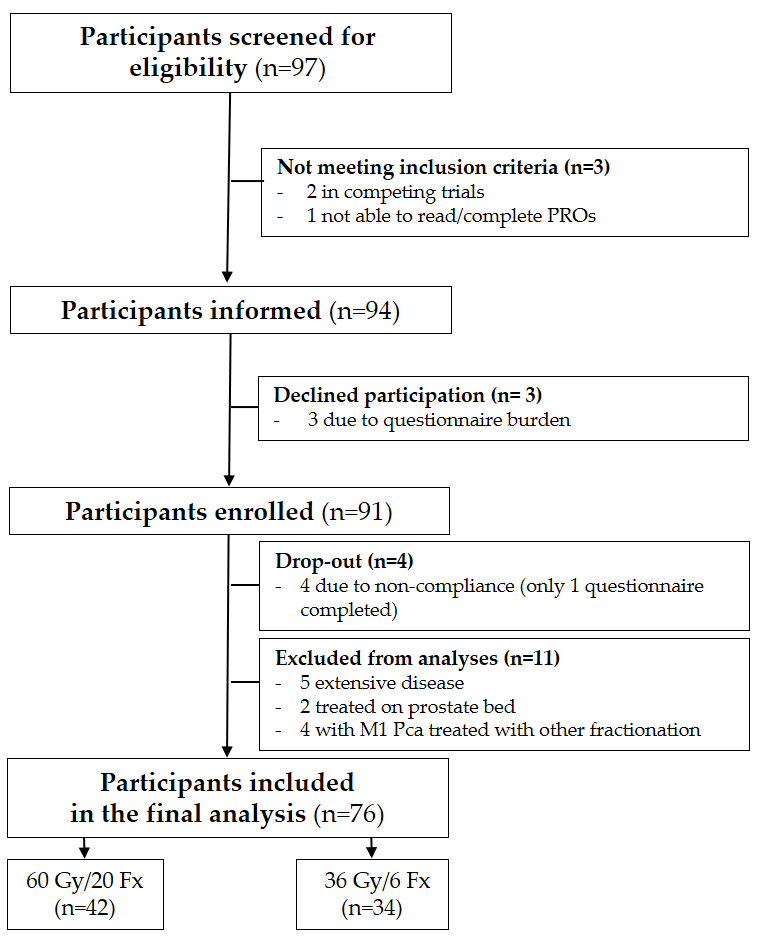


**Figure A.3.** Percentage of ePROs completed per patient in each group

**Table A.1**. Longitudinal prevalence of patient-reported symptoms with high symptom level (PRO-CTCAE 2-4, EORTC 3-4)

|  | Low-volume M1 PCa, 36 Gy/ 6 Fx (n=34) | | | | | | | | | |
| --- | --- | --- | --- | --- | --- | --- | --- | --- | --- | --- |
|  | *High symptom level^a^, n (% of responders at each time point)* | | | | | | | | | |
|  | Baseline | RT wk 1 | RT wk2 | FU wk1 | FU wk2 | FU wk3 | FU wk 4 | FU wk 8 | FU wk 12 | FU wk 24 |
| n responders | 32 | 34 | 33 | 29 | 25 | 27 | 26 | 28 | 29 | 28 |
| **Genitourinary** |  |  |  |  |  |  |  |  |  |  |
| Urinary frequency (F)^b^ | 25(74) | 29(85) | 30(88) | 24(71) | 22(65) | 20(59) | 18(53) | 22(65) | 19(56) | 16(47) |
| Urinary urge | 8(25) | 14(41) | 17(52) | 12(41) | 9(36) | 8(30) | 7(27) | 5(18) | 6(21) | 5(18) |
| Painful urination | 5(16) | 15(44) | 13 (39) | 8(28) | 7(28) | 6(22) | 7(27) | 3(11) | 2(7) | 1(4) |
| Difficulty emptying bladder (retention) | 3(9) | 10(29) | 12(36) | 8(28) | 7(28) | 5(19) | 5(19) | 2(7) | 3(10) | 3(11) |
| Frequent urination at night (nocturia) | 12(38) | 19(56) | 21(64) | 17(59) | 17(68) | 15(56) | 9(35) | 10(36) | 10(35) | 4 (14) |
| Urinary incontinence | 0 | 0 | 1(3) | 0 | 0 | 0 | 0 | 0 | 0 | 0 |
| **Gastrointestional** |  |  |  |  |  |  |  |  |  |  |
| Diarrhoea (F)^b^ | 2(6) | 3(9) | 4 (12) | 5(17) | 4 (16) | 3(11) | 2(8) | 3(11) | 1(4) | 1(4) |
| Abdominal pain | 2(6) | 2(6) | 1(3) | 1(3) | 0 | 1(4) | 1(4) | 5(18) | 3(10) | 3(11) |
| Nausea | 0 | 0 | 0 | 0 | 0 | 0 | 0 | 0 | 1(3) | 0 |
| Decreased appetite | 4 (13) | 2(6) | 3(9) | 1(3) | 1(4) | 1(4) | 1(4) | 1(4) | 2(7) | 1(4) |
| Constipation | 1(3) | 2(6) | 3(9) | 4 (14) | 3(12) | 4 (15) | 1(4) | 4 (14) | 3(10) | 2(7) |
| Difficulty controlling bowels | 0 | 2(6) | 2(6) | 3(10) | 0 | 1(4) | 0 | 1(4) | 0 | 0 |
| Unable empty bowels | 2(6) | 3(9) | 3(9) | 5(17) | 2(8) | 3(11) | 2(8) | 4(14) | 3(10) | 3(11) |
| Rectal pain/discomfort | 0 | 1(3) | 2(6) | 3(10) | 2(8) | 1(4) | 0 | 2(7) | 1(4) | 1(4) |
| Blood in stools | 0 | 0 | 0 | 1(3) | 0 | 0 | 0 | 0 | 0 | 0 |
| Bloated feeling in abdomen | 1(3) | 2(6) | 3(9) | 4 (14) | 3(12) | 1(4) | 1(4) | 4 (14) | 2(7) | 2(7) |
| **Other** |  |  |  |  |  |  |  |  |  |  |
| Fatigue | 13 (41) | 12(35) | 9(27) | 9(31) | 9(36) | 5(19) | 8(31) | 9(32) | 10(35) | 10(36) |
| Radiation skin reaction | 0 | 1(3) | 2(6) | 2(7) | 2(8) | 1(4) | 1(4) | 2(7) | 1(3) | 3(11) |
| Achieve and maintain erection | 29(94) |  |  |  |  |  | 23(88) | 25(89) | 26(93) | 26(93) |
| Decreased libido | 28(90) |  |  |  |  |  | 22(92) | 25(89) | 26(93) | 26(93) |
| RT = radiotherapy week. FU = Follow-up week  ^a^ (S) Severity score: PRO-CTCAE 2-4 (Moderate/severe/very severe), EORTC 3-4 (Quite a bit/very much). | | | | | |  |  |  |  |  |
| ^b^ (F) Frequency score: PRO-CTCAE 2-4: Occasionally/Frequently/Almost constantly | | | |  |  |  |  |  |  |  |

|  | Low-volume M1 PCa, 60 Gy/ 20 Fx (n=42) | | | | | | | | | | | |
| --- | --- | --- | --- | --- | --- | --- | --- | --- | --- | --- | --- | --- |
|  | *High symptom level^a^, n (% of responders at each time point)* | | | | | | | | | | | |
|  | BL | RT 1 | RT 2 | RT 3 | RT 4 | FU wk1 | FU wk2 | FU wk3 | FU wk 4 | FU wk 8 | FU wk 12 | FU wk 24 |
| n responders | 41 | 41 | 39 | 40 | 39 | 35 | 30 | 24 | 31 | 32 | 36 | 37 |
| **Genitourinary** |  |  |  |  |  |  |  |  |  |  |  |  |
| Urinary frequency (F)^b^ | 23(56) | 28(68) | 34(87) | 38(95) | 35(90) | 31(89) | 24(80) | 15(63) | 18(58) | 15(47) | 15(42) | 14(38) |
| Urinary urge | 10(24) | 10(24) | 15(39) | 19(48) | 21(54) | 20(57) | 13(43) | 10(42) | 13(42) | 10(31) | 8(22) | 6(16) |
| Painful urination | 2(5) | 6(15) | 12(31) | 14(35) | 13(33) | 13(37) | 12(40) | 7(29) | 5(16) | 3(9) | 2(6) | 0 |
| Difficulty emptying bladder (retention) | 3(7) | 9(22) | 11(28) | 14(35) | 14(36) | 13(37) | 8(27) | 5(21) | 7(23) | 3(9) | 2(6) | 4(11) |
| Frequent urination at night (nocturia) | 12(29) | 15(37) | 17(44) | 17(43) | 23(59) | 19(54) | 18(60) | 11(46) | 12(39) | 8(25) | 9(25) | 12(32) |
| Urinary incontinence | 1(2) | 1(2) | 1(3) | 2(5) | 1(3) | 1(3) | 1(3) | 1(4) | 2(6) | 1(3) | 0 | 0 |
| **Gastrointestional** |  |  |  |  |  |  |  |  |  |  |  |  |
| Diarrhoea (F)^b^ | 1(2) | 3(7) | 7(18) | 11(28) | 10(26) | 8(23) | 4(13) | 3(13) | 4(13) | 2(6) | 5(14) | 3(8) |
| Abdominal pain | 2(5) | 4(10) | 2(5) | 3(8) | 3(8) | 2(6) | 3(10) | 2(8) | 4(13) | 2(6) | 2(6) | 3(8) |
| Nausea | 1(2) | 1(2) | 1(3) | 0 | 0 | 0 | 0 | 0 | 0 | 0 | 0 | 1(3) |
| Decreased appetite | 2(5) | 1(2) | 2(5) | 3(8) | 4(10) | 4(11) | 3(10) | 2(8) | 2(6) | 0 | 0 | 2(5) |
| Constipation | 2(5) | 2(5) | 4(10) | 5(13) | 8(21) | 3(9) | 5(17) | 2(8) | 2(6) | 1(3) | 0 | 2(5) |
| Difficulty controlling bowels | 0 | 1(2) | 0 | 2(5) | 2(5) | 2(6) | 1(3) | 0 | 2(6) | 2(6) | 2(6) | 2(5) |
| Unable empty bowels | 3(8) | 5(12) | 6(15) | 7(18) | 8(21) | 6(17) | 4(13) | 3(13) | 3(10) | 1(3) | 1(3) | 2(5) |
| Rectal pain/discomfort | 1(2) | 0 | 1(3) | 3(8) | 5(13) | 3(9) | 4(13) | 1(4) | 2(6) | 1(3) | 1(3) | 1(3) |
| Blood in stools | 0 | 1(2) | 1(3) | 2(5) | 1(3) | 2(6) | 0 | 0 | 0 | 0 | 0 | 0 |
| Bloated feeling in abdomen | 3(7) | 2(5) | 2(5) | 2(5) | 2(5) | 5(14) | 3(10) | 2(8) | 1(3) | 2(6) | 4(11) | 3(8) |
| **Other** |  |  |  |  |  |  |  |  |  |  |  |  |
| Fatigue | 8(20) | 11(27) | 12(31) | 16(40) | 14(36) | 16(46) | 11(37) | 8(33) | 8(26) | 8(25) | 9(25) | 11(30) |
| Radiation skin reaction | 0 | 1(3) | 0 | 0 | 0 | 1(3) | 1(3) | 0 | 0 | 0 | 0 | 0 |
| Achieve and maintain erection | 25(66) |  |  |  |  |  |  |  | 23(74) | 18(56) | 22(61) | 19(53) |
| Decreased libido | 24(59) |  |  |  |  |  |  |  | 17(55) | 20(63) | 21(58) | 19(53) |
| RT = radiotherapy week. FU = Follow-up week  ^a^ (S) Severity score: PRO-CTCAE 2-4 (Moderate/severe/very severe), EORTC 3-4 (Quite a bit/very much). | | | | | |  |  |  |  |  |  |  |
| ^b^ (F) Frequency score: PRO-CTCAE 2-4: Occasionally/Frequently/Almost constantly | | | |  |  |  |  |  |  |  |  |  |

**STable A.2**. Linear Mixed Model results of changes in mean symptom scores relative to baseline for patients treated with MRgRT (n=76)

| LOW-VOLUME METASTATIC Pca (36 Gy/6 fx ) | | | | | |  | LOCALISED Pca (60 Gy/20 fx) | | | | | |
| --- | --- | --- | --- | --- | --- | --- | --- | --- | --- | --- | --- | --- |
|  |  | |  |  |  |  |  |  | |  |  |  |
| URINARY FREQUENCY |  | |  |  |  |  | **URINARY FREQUENCY** |  | |  |  |  |
|  | Estimate | | 95% CI | | *p-value* |  |  | Estimate | | 95% CI | | *p-value* |
| (Intercept) | 1.97 | |  |  |  |  | (Intercept) | 1.62 | |  |  |  |
| RT wk1 | 0.41 | | -0.10 | 0.91 | *0.245* |  | RT wk1 | 0.32 | | -0.18 | 0.83 | *0.629* |
|  |  | |  |  |  |  | **RT wk2** | **0.66** | | **0.15** | **1.18** | ***0.002*** |
|  |  | |  |  |  |  | **RT wk3** | **0.83** | | **0.33** | **1.34** | ***<.001*** |
| End of RT | **0.57** | | **0.06** | **1.08** | ***0.014*** |  | **End RT** | **0.83** | | **0.32** | **1.34** | ***<.001*** |
| FU wk1 | 0.40 | | -0.13 | 0.92 | *0.321* |  | **FU wk1** | **0.79** | | **0.26** | **1.32** | ***<.001*** |
| wk2 | 0.33 | | -0.23 | 0.88 | *0.678* |  | **wk2** | **0.70** | | **0.14** | **1.25** | ***0.002*** |
| wk3 | 0.20 | | -0.34 | 0.75 | *0.974* |  | wk3 | 0.32 | | -0.28 | 0.91 | *0.843* |
| wk4 | -0.16 | | -0.71 | 0.38 | *0.994* |  | wk4 | 0.40 | | -0.15 | 0.95 | *0.425* |
| wk8 | -0.06 | | -0.59 | 0.47 | *1.000* |  | wk8 | -0.10 | | -0.64 | 0.45 | *1.000* |
| wk12 | -0.17 | | -0.70 | 0.36 | *0.990* |  | wk12 | -0.03 | | -0.55 | 0.50 | *1.000* |
| wk24 | -0.33 | | -0.86 | 0.20 | *0.615* |  | wk24 | -0.15 | | -0.67 | 0.38 | *0.999* |
|  |  | |  |  |  |  |  |  | |  |  |  |
| URINARY URGE | Estimate | | 95% CI | | *p-value* |  | **URINARY URGE** | Estimate | | 95% CI | | *p-value* |
| (Intercept) | 2.17 | |  |  |  |  | (Intercept) | 1.89 | |  |  |  |
| RT wk1 | 0.16 | | -0.29 | 0.61 | *0.980* |  | RT wk1 | 0.15 | | -0.26 | 0.55 | *0.989* |
|  |  | |  |  |  |  | RT wk2 | 0.37 | | -0.03 | 0.78 | *0.110* |
|  |  | |  |  |  |  | **RT wk3** | **0.60** | | **0.20** | **1.01** | ***<.001*** |
| End of RT | 0.32 | | -0.13 | 0.78 | *0.408* |  | **End RT** | **0.58** | | **0.18** | **0.99** | ***<.001*** |
| FU wk1 | 0.38 | | -0.09 | 0.85 | *0.222* |  | **FU wk1** | **0.69** | | **0.27** | **1.11** | ***<.001*** |
| wk2 | 0.23 | | -0.26 | 0.72 | *0.900* |  | **wk2** | **0.52** | | **0.08** | **0.96** | ***0.006*** |
| wk3 | 0.20 | | -0.29 | 0.68 | *0.952* |  | wk3 | 0.40 | | -0.07 | 0.88 | *0.179* |
| wk4 | 0.00 | | -0.49 | 0.49 | *1.000* |  | **wk4** | **0.47** | | **0.04** | **0.91** | ***0.019*** |
| wk8 | -0.09 | | -0.56 | 0.38 | *1.000* |  | wk8 | 0.23 | | -0.20 | 0.66 | *0.821* |
| wk12 | -0.14 | | -0.61 | 0.33 | *0.994* |  | wk12 | 0.19 | | -0.22 | 0.61 | *0.934* |
| wk24 | -0.25 | | -0.73 | 0.22 | *0.787* |  | wk24 | 0.02 | | -0.40 | 0.43 | *1.000* |
| LOW-VOLUME METASTATIC Pca (36 Gy/6 fx ) | | | | | |  | **LOCALISED Pca (60 Gy/20 fx)** | | | | | |
|  |  | |  |  |  |  |  |  | |  |  |  |
| PAINFUL URINATION | Estimate | | 95% CI | | *p-value* |  | **PAINFUL URINATION** | Estimate | | 95% CI | | *p-value* |
| (Intercept) | 0.50 | |  |  |  |  | (Intercept) | 0.14 | |  |  |  |
| RT wk1 | **0.86** | | **0.33** | **1.38** | ***<.001*** |  | RT wk1 | 0.39 | | -0.10 | 0.87 | *0.274* |
|  |  | |  |  |  |  | **RT wk2** | **0.94** | | **0.45** | **1.43** | ***<.001*** |
|  |  | |  |  |  |  | **RT wk3** | **1.15** | | **0.66** | **1.64** | ***<.001*** |
| End of RT | **0.78** | | **0.25** | **1.30** | ***<.001*** |  | **End RT** | **1.08** | | **0.59** | **1.57** | ***<.001*** |
| FU wk1 | **0.69** | | **0.15** | **1.24** | ***0.002*** |  | **FU wk1** | **1.10** | | **0.60** | **1.61** | ***<.001*** |
| wk2 | 0.49 | | -0.08 | 1.06 | *0.158* |  | **wk2** | **1.04** | | **0.51** | **1.57** | ***<.001*** |
| wk3 | 0.38 | | -0.19 | 0.94 | *0.506* |  | **wk3** | **0.76** | | **0.19** | **1.33** | ***0.001*** |
| wk4 | 0.24 | | -0.32 | 0.80 | *0.935* |  | wk4 | 0.44 | | -0.09 | 0.97 | *0.208* |
| wk8 | -0.18 | | -0.73 | 0.37 | *0.990* |  | wk8 | 0.21 | | -0.31 | 0.73 | *0.976* |
| wk12 | 0.00 | | -0.55 | 0.54 | *1.000* |  | wk12 | 0.19 | | -0.31 | 0.70 | *0.982* |
| wk24 | -0.28 | | -0.82 | 0.27 | *0.844* |  | wk24 | 0.02 | | -0.48 | 0.52 | *1.000* |
|  |  | |  |  |  |  |  |  | |  |  |  |
|  |  | |  |  |  |  |  |  | |  |  |  |
| DIFFICULTY EMPTYING BLADDER (RETENTION) | Estimate | | 95% CI | | *p-value* |  | **DIFFICULTY EMPTYING BLADDER (RETENTION)** | Estimate | | 95% CI | | *p-value* |
| (Intercept) | 1.74 | |  |  |  |  | (Intercept) | 1.46 | |  |  |  |
| RT wk1 | 0.32 | | -0.13 | 0.77 | *0.399* |  | **RT wk1** | **0.44** | | **0.02** | **0.87** | ***0.028*** |
|  |  | |  |  |  |  | **RT wk2** | **0.60** | | **0.17** | **1.02** | ***<.001*** |
|  |  | |  |  |  |  | **RT wk3** | **0.74** | | **0.32** | **1.17** | ***<.001*** |
| End of RT | **0.46** | | **0.01** | **0.91** | ***0.042*** |  | **End RT** | **0.76** | | **0.33** | **1.18** | ***<.001*** |
| FU wk1 | 0.32 | | -0.14 | 0.79 | *0.454* |  | **FU wk1** | **0.84** | | **0.40** | **1.28** | ***<.001*** |
| wk2 | **0.55** | | **0.07** | **1.04** | ***0.013*** |  | **wk2** | **0.59** | | **0.13** | **1.06** | ***0.002*** |
| wk3 | 0.19 | | -0.29 | 0.67 | *0.960* |  | **wk3** | **0.52** | | **0.03** | **1.02** | ***0.028*** |
| wk4 | 0.06 | | -0.43 | 0.54 | *1.000* |  | wk4 | 0.34 | | -0.12 | 0.80 | *0.392* |
| wk8 | -0.07 | | -0.54 | 0.40 | *1.000* |  | wk8 | 0.14 | | -0.31 | 0.59 | *0.997* |
| wk12 | -0.02 | | -0.49 | 0.44 | *1.000* |  | wk12 | 0.19 | | -0.24 | 0.63 | *0.950* |
| wk24 | -0.12 | | -0.58 | 0.35 | *0.999* |  | wk24 | 0.10 | | -0.33 | 0.54 | *1.000* |
|  |  | |  |  |  |  |  |  | |  |  |  |
|  |  | |  |  |  |  |  |  | |  |  |  |
| LOW-VOLUME METASTATIC Pca (36 Gy/6 fx ) | | | | | |  | **LOCALISED Pca (60 Gy/20 fx)** | | | | | |
|  |  | |  |  |  |  |  |  | |  |  |  |
| FREQUENT URINATION AT NIGHT (NOCTURIA) | Estimate | | 95% CI | | *p-value* |  | **FREQUENT URINATION AT NIGHT (NOCTURIA)** | Estimate | | 95% CI | | *p-value* |
| (Intercept) | 2.31 | | 2.04 | 2.57 |  |  | (Intercept) | 2.23 | |  |  |  |
| RT wk1 | 0.35 | | -0.10 | 0.81 | *0.281* |  | RT wk1 | -0.02 | | -0.38 | 0.34 | *1.000* |
|  |  | |  |  |  |  | RT wk2 | 0.21 | | -0.16 | 0.57 | *0.779* |
|  |  | |  |  |  |  | RT wk3 | 0.25 | | -0.11 | 0.62 | *0.467* |
| End of RT | **0.46** | | **0.00** | **0.92** | ***0.046*** |  | **End RT** | **0.39** | | **0.02** | **0.75** | ***0.026*** |
| FU wk1 | 0.32 | | -0.15 | 0.79 | *0.493* |  | **FU wk1** | **0.43** | | **0.05** | **0.81** | ***0.011*** |
| wk2 | 0.38 | | -0.12 | 0.88 | *0.300* |  | wk2 | 0.39 | | -0.01 | 0.78 | *0.061* |
| wk3 | 0.35 | | -0.13 | 0.84 | *0.384* |  | wk3 | 0.21 | | -0.21 | 0.64 | *0.894* |
| wk4 | -0.03 | | -0.52 | 0.46 | *1.000* |  | wk4 | 0.17 | | -0.22 | 0.56 | *0.959* |
| wk8 | -0.04 | | -0.52 | 0.44 | *1.000* |  | wk8 | -0.12 | | -0.51 | 0.26 | *0.997* |
| wk12 | -0.07 | | -0.54 | 0.40 | *1.000* |  | wk12 | -0.07 | | -0.45 | 0.30 | *1.000* |
| wk24 | -0.09 | | -0.57 | 0.38 | *1.000* |  | wk24 | -0.04 | | -0.41 | 0.34 | *1.000* |
|  |  | |  |  |  |  |  |  | |  |  |  |
|  |  | |  |  |  |  |  |  | |  |  |  |
| URINARY INCONTINENCE | Estimate | | 95% CI | | *p-value* |  | **URINARY INCONTINENCE** | Estimate | | 95% CI | | *p-value* |
| (Intercept) | 1.18 | |  |  |  |  | (Intercept) | 1.18 | |  |  |  |
| RT wk1 | -0.03 | | -0.27 | 0.21 | *1.000* |  | RT wk1 | 0.01 | | -0.24 | 0.25 | *1.000* |
|  |  | |  |  |  |  | RT wk2 | 0.01 | | -0.24 | 0.25 | *1.000* |
|  |  | |  |  |  |  | RT wk3 | 0.11 | | -0.14 | 0.35 | *0.959* |
| End of RT | 0.08 | | -0.16 | 0.33 | *0.982* |  | End RT | 0.01 | | -0.24 | 0.26 | *1.000* |
| FU wk1 | 0.09 | | -0.16 | 0.34 | *0.972* |  | FU wk1 | 0.07 | | -0.18 | 0.33 | *0.998* |
| wk2 | 0.04 | | -0.22 | 0.30 | *1.000* |  | wk2 | 0.13 | | -0.14 | 0.40 | *0.910* |
| wk3 | -0.01 | | -0.27 | 0.25 | *1.000* |  | wk3 | 0.08 | | -0.21 | 0.37 | *0.999* |
| wk4 | 0.02 | | -0.24 | 0.28 | *1.000* |  | wk4 | 0.15 | | -0.12 | 0.41 | *0.819* |
| wk8 | -0.05 | | -0.30 | 0.20 | *1.000* |  | wk8 | 0.07 | | -0.19 | 0.33 | *0.999* |
| wk12 | -0.03 | | -0.28 | 0.21 | *1.000* |  | wk12 | 0.09 | | -0.16 | 0.35 | *0.990* |
| wk24 | 0.02 | | -0.23 | 0.28 | *1.000* |  | wk24 | 0.06 | | -0.19 | 0.31 | *1.000* |
|  |  | |  |  |  |  |  |  | |  |  |  |
|  |  | |  |  |  |  |  |  | |  |  |  |
| LOW-VOLUME METASTATIC Pca (36 Gy/6 fx ) | | | | | |  | **LOCALISED Pca (60 Gy/20 fx)** | | | | | |
|  |  | |  |  |  |  |  |  | |  |  |  |
| DIARRHOEA | Estimate | | 95% CI | | *p-value* |  | **DIARRHOEA** | Estimate | | 95% CI | | *p-value* |
| (Intercept) | 0.36 | |  |  |  |  | (Intercept) | 0.31 | |  |  |  |
| RT wk1 | 0.12 | | -0.29 | 0.54 | *0.994* |  | RT wk1 | 0.17 | | -0.28 | 0.62 | *0.984* |
|  |  | |  |  |  |  | RT wk2 | 0.42 | | -0.04 | 0.87 | *0.115* |
|  |  | |  |  |  |  | **RT wk3** | **0.64** | | **0.19** | **1.09** | ***<.001*** |
| End of RT | 0.30 | | -0.11 | 0.72 | *0.377* |  | **End RT** | **0.55** | | **0.09** | **1.00** | ***0.006*** |
| FU wk1 | **0.45** | | **0.02** | **0.88** | ***0.029*** |  | **FU wk1** | **0.53** | | **0.06** | **1.00** | ***0.014*** |
| wk2 | 0.27 | | -0.18 | 0.72 | *0.680* |  | wk2 | 0.20 | | -0.30 | 0.69 | *0.979* |
| wk3 | 0.16 | | -0.29 | 0.60 | *0.981* |  | wk3 | 0.12 | | -0.42 | 0.65 | *1.000* |
| wk4 | -0.16 | | -0.61 | 0.28 | *0.976* |  | wk4 | 0.21 | | -0.28 | 0.70 | *0.958* |
| wk8 | 0.05 | | -0.38 | 0.49 | *1.000* |  | wk8 | 0.09 | | -0.40 | 0.57 | *1.000* |
| wk12 | -0.01 | | -0.44 | 0.42 | *1.000* |  | wk12 | 0.17 | | -0.30 | 0.64 | *0.990* |
| wk24 | -0.06 | | -0.49 | 0.38 | *1.000* |  | wk24 | 0.21 | | -0.25 | 0.68 | *0.938* |
|  |  | |  |  |  |  |  |  | |  |  |  |
|  |  | |  |  |  |  |  |  | |  |  |  |
| ABDOMINAL PAIN | Estimate | | 95% CI | | *p-value* |  | **ABDOMINAL PAIN** | Estimate | | 95% CI | | *p-value* |
| (Intercept) | 1.00 | |  |  |  |  | (Intercept) | 1.11 | |  |  |  |
| RT wk1 | 0.04 | | -7.14 | 0.79 | *1.000* |  | RT wk1 | 0.10 | | -0.52 | 0.72 | *1.000* |
|  |  | |  |  |  |  | RT wk2 | 0.08 | | -0.56 | 0.73 | *1.000* |
|  |  | |  |  |  |  | RT wk3 | 0.17 | | -0.46 | 0.79 | *0.999* |
| End of RT | -0.10 | | -0.85 | 0.68 | *1.000* |  | End RT | 0.08 | | -0.54 | 0.70 | *1.000* |
| FU wk1 | 0.12 | | -0.60 | 0.85 | *1.000* |  | FU wk1 | 0.11 | | -0.53 | 0.75 | *1.000* |
| wk2 | -0.08 | | -1.00 | 0.85 | *1.000* |  | wk2 | 0.23 | | -0.44 | 0.90 | *0.992* |
| wk3 | 0.05 | | -0.90 | 1.01 | *1.000* |  | wk3 | 0.12 | | -0.63 | 0.88 | *1.000* |
| wk4 | 0.00 | | -0.84 | 0.84 | *1.000* |  | wk4 | 0.32 | | -0.37 | 1.01 | *0.919* |
| wk8 | 0.31 | | -0.40 | 1.02 | *0.919* |  | wk8 | 0.04 | | -0.65 | 0.73 | *1.000* |
| wk12 | 0.20 | | -0.57 | 0.97 | *0.997* |  | wk12 | 0.07 | | -0.64 | 0.79 | *1.000* |
| wk24 | 0.00 | | -0.75 | 0.74 | *1.000* |  | wk24 | 0.12 | | -0.58 | 0.81 | *1.000* |
|  |  | |  |  |  |  |  |  | |  |  |  |
|  |  | |  |  |  |  |  |  | |  |  |  |
| LOW-VOLUME METASTATIC Pca (36 Gy/6 fx ) | | | | | |  | **LOCALISED Pca (60 Gy/20 fx)** | | | | | |
|  |  | |  |  |  |  |  |  | |  |  |  |
| NAUSEA | Estimate | | 95% CI | | *p-value* |  | **NAUSEA** | Estimate | | 95% CI | | *p-value* |
| (Intercept) | 0.21 | |  |  |  |  | (Intercept) | 0.13 | |  |  |  |
| RT wk1 | 0.00 | | -0.16 | 0.16 | *0.982* |  | RT wk1 | 0.01 | | -0.11 | 0.14 | *0.838* |
|  |  | |  |  |  |  | RT wk2 | -0.01 | | -0.14 | 0.12 | *0.883* |
|  |  | |  |  |  |  | RT wk3 | -0.14 | | -0.26 | -0.01 | *0.035* |
| End of RT | 0.03 | | -0.13 | 0.19 | *0.691* |  | End RT | -0.03 | | -0.16 | 0.10 | *0.626* |
| FU wk1 | -0.05 | | -0.22 | 0.12 | *0.562* |  | FU wk1 | -0.06 | | -0.19 | 0.07 | *0.345* |
| wk2 | -0.01 | | -0.18 | 0.17 | *0.951* |  | wk2 | 0.01 | | -0.13 | 0.15 | *0.879* |
| wk3 | -0.07 | | -0.24 | 0.10 | *0.435* |  | wk3 | -0.01 | | -0.16 | 0.14 | *0.873* |
| wk4 | -0.03 | | -0.20 | 0.15 | *0.764* |  | wk4 | -0.01 | | -0.15 | 0.13 | *0.878* |
| wk8 | -0.08 | | -0.25 | 0.08 | *0.326* |  | wk8 | -0.05 | | -0.18 | 0.09 | *0.477* |
| wk12 | 0.09 | | -0.07 | 0.26 | *0.281* |  | wk12 | -0.06 | | -0.19 | 0.07 | *0.375* |
| wk24 | -0.07 | | -0.24 | 0.10 | *0.437* |  | wk24 | -0.05 | | -0.18 | 0.08 | *0.429* |
|  | | | | | |  |  | | | | | |
|  | | | | | |  |  | | | | | |
| CONSTIPATION | Estimate | | 95% CI | | *p-value* |  | **CONSTIPATION** | Estimate | | 95% CI | | *p-value* |
| (Intercept) | 0.29 | |  |  |  |  | (Intercept) | 0.28 | |  |  |  |
| RT wk1 | 0.05 | | -0.19 | 0.29 | *0.693* |  | RT wk1 | -0.11 | | -0.35 | 0.13 | *0.362* |
|  |  | |  |  |  |  | RT wk2 | 0.09 | | -0.15 | 0.33 | *0.468* |
|  |  | |  |  |  |  | RT wk3 | 0.14 | | -0.10 | 0.38 | *0.268* |
| End of RT | 0.08 | | -0.16 | 0.32 | *0.505* |  | **End RT** | **0.32** | | **0.08** | **0.57** | ***0.009*** |
| FU wk1 | 0.05 | | -0.20 | 0.30 | *0.701* |  | FU wk1 | 0.03 | | -0.22 | 0.28 | *0.825* |
| wk2 | **0.43** | | **0.16** | **0.69** | ***0.002*** |  | wk2 | 0.24 | | -0.02 | 0.50 | *0.073* |
| wk3 | 0.24 | | -0.02 | 0.50 | *0.068* |  | wk3 | 0.02 | | -0.26 | 0.30 | *0.895* |
| wk4 | -0.04 | | -0.30 | 0.22 | *0.761* |  | wk4 | -0.05 | | -0.31 | 0.21 | *0.706* |
| wk8 | 0.17 | | -0.08 | 0.43 | *0.178* |  | wk8 | -0.13 | | -0.39 | 0.12 | *0.313* |
| wk12 | 0.00 | | -0.25 | 0.25 | *0.987* |  | wk12 | -0.23 | | -0.48 | 0.02 | *0.072* |
| wk24 | 0.03 | | -0.22 | 0.28 | *0.816* |  | wk24 | -0.04 | | -0.29 | 0.20 | *0.731* |
|  |  | |  |  |  |  |  |  | |  |  |  |
|  | | | |  |  |  |  | | | |  |  |
| LOW-VOLUME METASTATIC Pcaj (36 Gy/6 fx ) | | | | | |  | **LOCALISED Pca (60 Gy/20 fx)** | | | | | |
|  | | | |  |  |  |  | | | |  |  |
| DIFFICULTY CONTROLLING BOWELS | Estimate | 95% CI | | | *p-value* |  | **DIFFICULTY CONTROLLING BOWELS** | Estimate | 95% CI | | | *p-value* |
| (Intercept) | 1.09 | |  |  |  |  | (Intercept) | 1.07 | |  |  |  |
| RT wk1 | 0.24 | | -0.07 | 0.55 | *0.275* |  | RT wk1 | 0.09 | | -0.22 | 0.39 | *0.999* |
|  |  | |  |  |  |  | RT wk2 | 0.08 | | -0.23 | 0.38 | *1.000* |
|  |  | |  |  |  |  | **RT wk3** | **0.37** | | **0.07** | **0.68** | ***0.004*** |
| End of RT | 0.24 | | -0.07 | 0.54 | *0.313* |  | End RT | 0.30 | | 0.00 | 0.61 | *0.055* |
| FU wk1 | **0.40** | | **0.08** | **0.72** | ***0.004*** |  | FU wk1 | 0.24 | | -0.08 | 0.55 | *0.369* |
| wk2 | 0.09 | | -0.25 | 0.42 | *0.998* |  | wk2 | 0.12 | | -0.21 | 0.45 | *0.987* |
| wk3 | 0.15 | | -0.18 | 0.49 | *0.894* |  | wk3 | 0.10 | | -0.26 | 0.46 | *0.999* |
| wk4 | -0.01 | | -0.34 | 0.32 | *1.000* |  | wk4 | 0.20 | | -0.12 | 0.53 | *0.666* |
| wk8 | 0.04 | | -0.28 | 0.37 | *1.000* |  | wk8 | 0.12 | | -0.20 | 0.45 | *0.986* |
| wk12 | 0.05 | | -0.27 | 0.37 | *1.000* |  | wk12 | 0.20 | | -0.11 | 0.52 | *0.590* |
| wk24 | 0.18 | | -0.14 | 0.51 | *0.728* |  | wk24 | 0.14 | | -0.17 | 0.45 | *0.948* |
|  | | | | | |  |  | | | | | |
|  | | | | | |  |  | | | | | |
| UNABLE EMPTY BOWELS | Estimate | | 95% CI | | *p-value* |  | **UNABLE EMPTY BOWELS** | Estimate | | 95% CI | | *p-value* |
| (Intercept) | 1.40 | |  |  |  |  | (Intercept) | 1.31 | |  |  |  |
| RT wk1 | 0.06 | | -0.26 | 0.40 | *1.000* |  | RT wk1 | 0.30 | | -0.09 | 0.70 | *0.326* |
|  |  | |  |  |  |  | **RT wk2** | **0.45** | | **0.05** | **0.85** | ***0.011*** |
|  |  | |  |  |  |  | **RT wk3** | **0.56** | | **0.16** | **0.96** | ***<.001*** |
| End of RT | 0.10 | | -0.22 | 0.43 | *0.992* |  | **End RT** | **0.62** | | **0.22** | **1.02** | ***<.001*** |
| FU wk1 | 0.33 | | -0.01 | 0.67 | *0.065* |  | **FU wk1** | **0.48** | | **0.07** | **0.89** | ***0.008*** |
| wk2 | 0.13 | | -0.22 | 0.49 | *0.973* |  | wk2 | 0.39 | | -0.04 | 0.83 | *0.114* |
| wk3 | 0.05 | | -0.31 | 0.40 | *1.000* |  | wk3 | 0.28 | | -0.18 | 0.75 | *0.684* |
| wk4 | 0.09 | | -0.26 | 0.44 | *0.999* |  | wk4 | 0.22 | | -0.21 | 0.65 | *0.864* |
| wk8 | 0.19 | | -0.16 | 0.53 | *0.771* |  | wk8 | 0.02 | | -0.41 | 0.44 | *1.000* |
| wk12 | 0.10 | | -0.24 | 0.44 | *0.996* |  | wk12 | 0.07 | | -0.34 | 0.48 | *1.000* |
| wk24 | 0.13 | | -0.21 | 0.48 | *0.963* |  | wk24 | 0.14 | | -0.27 | 0.55 | *0.994* |
|  |  | |  |  |  |  |  |  | |  |  |  |
|  |  | |  |  |  |  |  |  | |  |  |  |
| LOW-VOLUME METASTATIC Pca (36 Gy/6 fx ) | | | | | |  | **LOCALISED Pca (60 Gy/20 fx)** | | | | | |
|  |  | |  | |  |  |  |  | |  | |  |
| RECTAL PAIN/DISCOMFORT | Estimate | | 95% CI | | *p-value* |  | **RECTAL PAIN/DISCOMFORT** | Estimate | | 95% CI | | *p-value* |
| (Intercept) | 1.08 | |  |  |  |  | (Intercept) | 1.18 | |  |  |  |
| RT wk1 | 0.22 | | -0.11 | 0.56 | *0.520* |  | RT wk1 | -0.04 | | -0.38 | 0.30 | *1.000* |
|  |  | |  |  |  |  | RT wk2 | 0.09 | | -0.26 | 0.43 | *1.000* |
|  |  | |  |  |  |  | RT wk3 | 0.26 | | -0.08 | 0.60 | *0.327* |
| End of RT | 0.30 | | -0.04 | 0.64 | *0.127* |  | End RT | 0.33 | | -0.02 | 0.67 | *0.082* |
| FU wk1 | **0.41** | | **0.06** | **0.75** | ***0.009*** |  | FU wk1 | 0.27 | | -0.09 | 0.62 | *0.349* |
| wk2 | 0.36 | | 0.00 | 0.73 | *0.054* |  | wk2 | 0.24 | | -0.14 | 0.61 | *0.637* |
| wk3 | 0.26 | | -0.10 | 0.62 | *0.405* |  | wk3 | 0.14 | | -0.26 | 0.54 | *0.993* |
| wk4 | 0.10 | | -0.27 | 0.46 | *0.998* |  | wk4 | 0.01 | | -0.36 | 0.38 | *1.000* |
| wk8 | 0.23 | | -0.12 | 0.58 | *0.531* |  | wk8 | -0.05 | | -0.41 | 0.32 | *1.000* |
| wk12 | 0.12 | | -0.22 | 0.47 | *0.980* |  | wk12 | -0.02 | | -0.38 | 0.33 | *1.000* |
| wk24 | 0.06 | | -0.29 | 0.42 | *1.000* |  | wk24 | 0.01 | | -0.34 | 0.36 | *1.000* |
|  | | | | | |  |  | | | | | |
|  | | | | | |  |  | | | | | |
| BLOOD IN STOOL | Estimate | | 95% CI | | *p-value* |  | **BLOOD IN STOOL** | Estimate | | 95% CI | | *p-value* |
| (Intercept) | 1.00 | |  |  |  |  | (Intercept) | 1.00 | |  |  |  |
| RT wk1 | 0.03 | | -0.11 | 0.17 | *1.000* |  | RT wk1 | 0.10 | | -0.09 | 0.30 | *0.860* |
|  |  | |  |  |  |  | RT wk2 | 0.08 | | -0.12 | 0.27 | *0.980* |
|  |  | |  |  |  |  | RT wk3 | 0.15 | | -0.04 | 0.35 | *0.306* |
| End of RT | 0.06 | | -0.08 | 0.21 | *0.943* |  | End RT | 0.15 | | -0.04 | 0.35 | *0.294* |
| FU wk1 | 0.14 | | -0.01 | 0.29 | *0.086* |  | FU wk1 | 0.15 | | -0.05 | 0.36 | *0.341* |
| wk2 | 0.04 | | -0.12 | 0.20 | *0.998* |  | wk2 | 0.06 | | -0.15 | 0.27 | *0.999* |
| wk3 | 0.00 | | -0.15 | 0.16 | *1.000* |  | wk3 | 0.05 | | -0.17 | 0.28 | *1.000* |
| wk4 | 0.04 | | -0.12 | 0.19 | *0.999* |  | wk4 | -0.02 | | -0.23 | 0.19 | *1.000* |
| wk8 | 0.00 | | -0.15 | 0.15 | *1.000* |  | wk8 | 0.01 | | -0.19 | 0.22 | *1.000* |
| wk12 | 0.00 | | -0.15 | 0.15 | *1.000* |  | wk12 | 0.08 | | -0.12 | 0.28 | *0.978* |
| wk24 | 0.00 | | -0.15 | 0.15 | *1.000* |  | wk24 | 0.09 | | -0.11 | 0.29 | *0.953* |
|  |  | |  |  |  |  |  |  | |  |  |  |
|  |  | |  |  |  |  |  |  | |  |  |  |
| LOW-VOLUME METASTATIC Pca (36 Gy/6 fx ) | | | | | |  | **LOCALISED Pca (60 Gy/20 fx)** | | | | | |
|  |  | |  | |  |  |  |  | |  | |  |
| BLOATING | Estimate | | 95% CI | | *p-value* |  | **BLOATING** | Estimate | | 95% CI | | *p-value* |
| (Intercept) | 1.32 | |  |  |  |  | (Intercept) | 1.36 | |  |  |  |
| RT wk1 | -0.01 | | -0.36 | 0.35 | *1.000* |  | RT wk1 | -0.05 | | -0.38 | 0.29 | *1.000* |
|  |  | |  |  |  |  | RT wk2 | 0.21 | | -0.13 | 0.55 | *0.681* |
|  |  | |  |  |  |  | RT wk3 | 0.15 | | -0.19 | 0.48 | *0.951* |
| End of RT | 0.16 | | -0.19 | 0.51 | *0.914* |  | End RT | 0.10 | | -0.23 | 0.44 | *0.997* |
| FU wk1 | 0.30 | | -0.06 | 0.67 | *0.210* |  | FU wk1 | 0.23 | | -0.12 | 0.58 | *0.549* |
| wk2 | 0.19 | | -0.20 | 0.57 | *0.873* |  | wk2 | 0.00 | | -0.37 | 0.36 | *1.000* |
| wk3 | 0.17 | | -0.21 | 0.55 | *0.911* |  | wk3 | 0.08 | | -0.32 | 0.47 | *1.000* |
| wk4 | 0.02 | | -0.36 | 0.40 | *1.000* |  | wk4 | 0.01 | | -0.35 | 0.37 | *1.000* |
| wk8 | 0.08 | | -0.29 | 0.45 | *0.999* |  | wk8 | -0.06 | | -0.42 | 0.30 | *1.000* |
| wk12 | 0.03 | | -0.33 | 0.40 | *1.000* |  | wk12 | -0.04 | | -0.39 | 0.30 | *1.000* |
| wk24 | 0.12 | | -0.25 | 0.49 | *0.988* |  | wk24 | 0.02 | | -0.32 | 0.37 | *1.000* |
|  |  | |  |  |  |  |  |  | |  |  |  |
|  |  | |  |  |  |  |  |  | |  |  |  |
| FATIGUE | Estimate | | 95% CI | | *p-value* |  | **FATIGUE** | Estimate | | 95% CI | | *p-value* |
| (Intercept) | 1.21 | |  |  |  |  | (Intercept) | 0.75 | |  |  |  |
| RT wk1 | 0.03 | | -0.41 | 0.46 | *1.000* |  | RT wk1 | 0.21 | | -0.24 | 0.66 | *0.933* |
|  |  | |  |  |  |  | RT wk2 | 0.42 | | -0.04 | 0.87 | *0.111* |
|  |  | |  |  |  |  | **RT wk3** | **0.61** | | **0.16** | **1.06** | ***0.001*** |
| End of RT | 0.06 | | -0.38 | 0.49 | *1.000* |  | **End RT** | **0.49** | | **0.03** | **0.94** | ***0.025*** |
| FU wk1 | -0.07 | | -0.53 | 0.38 | *1.000* |  | **FU wk1** | **0.62** | | **0.15** | **1.09** | ***0.001*** |
| wk2 | 0.07 | | -0.41 | 0.54 | *1.000* |  | **wk2** | **0.49** | | **0.00** | **0.99** | ***0.048*** |
| wk3 | -0.19 | | -0.66 | 0.28 | *0.954* |  | wk3 | 0.27 | | -0.26 | 0.80 | *0.886* |
| wk4 | -0.16 | | -0.63 | 0.31 | *0.983* |  | wk4 | 0.32 | | -0.17 | 0.81 | *0.567* |
| wk8 | -0.15 | | -0.61 | 0.30 | *0.988* |  | wk8 | -0.03 | | -0.52 | 0.45 | *1.000* |
| wk12 | -0.12 | | -0.57 | 0.33 | *0.998* |  | wk12 | 0.15 | | -0.31 | 0.62 | *0.996* |
| wk24 | -0.01 | | -0.46 | 0.45 | *1.000* |  | wk24 | 0.26 | | -0.20 | 0.72 | *0.795* |
|  |  | |  |  |  |  |  |  | |  |  |  |
|  |  | |  |  |  |  |  |  | |  |  |  |
| LOW-VOLUME METASTATIC Pca (36 Gy/6 fx ) | | | | | |  | **LOCALISED Pca (60 Gy/20 fx)** | | | | | |
|  |  | |  |  |  |  |  |  | |  |  |  |
| APPETITE | Estimate | | 95% CI | | *p-value* |  | **APPETITE** | Estimate | | 95% CI | | *p-value* |
| (Intercept) | 0.36 | |  |  |  |  | (Intercept) | 0.24 | |  |  |  |
| RT wk1 | -0.05 | | -0.26 | 0.16 | *0.651* |  | RT wk1 | -0.05 | | -0.21 | 0.11 | *0.536* |
|  |  | |  |  |  |  | RT wk2 | 0.09 | | -0.08 | 0.25 | *0.293* |
|  |  | |  |  |  |  | RT wk3 | 0.01 | | -0.15 | 0.17 | *0.899* |
| End of RT | -0.09 | | -0.30 | 0.11 | *0.374* |  | End RT | 0.13 | | -0.04 | 0.29 | *0.133* |
| FU wk1 | -0.13 | | -0.35 | 0.09 | *0.245* |  | FU wk1 | 0.08 | | -0.09 | 0.25 | *0.375* |
| wk2 | -0.09 | | -0.31 | 0.14 | *0.457* |  | wk2 | -0.01 | | -0.19 | 0.17 | *0.917* |
| wk3 | -0.18 | | -0.40 | 0.04 | *0.115* |  | wk3 | -0.01 | | -0.21 | 0.18 | *0.884* |
| wk4 | **-0.27** | | **-0.50** | **-0.05** | ***0.017*** |  | wk4 | -0.15 | | -0.33 | 0.03 | *0.097* |
| wk8 | -0.13 | | -0.34 | 0.09 | *0.261* |  | wk8 | -0.16 | | -0.33 | 0.02 | *0.079* |
| wk12 | -0.04 | | -0.26 | 0.18 | *0.720* |  | wk12 | -0.16 | | -0.33 | 0.00 | *0.058* |
| wk24 | -0.15 | | -0.37 | 0.07 | *0.178* |  | wk24 | 0.01 | | -0.16 | 0.18 | *0.917* |

**Table A.3**. Changes in EQ-5D-5L dimension responses from baseline to follow-up week 12 and to follow-up week 24

|  | Week 12 (n=56) | | | | Week 24 (n=47) | | | |
| --- | --- | --- | --- | --- | --- | --- | --- | --- |
|  | M1 Pca | | Localised Pca | | M1 Pca | | Localised Pca | |
| **Mobility** | n | % | n | % | n | % | n | % |
| No change | 15 | 62.5 | 27 | 84.4 | 12 | 57.1 | 21 | 80.8 |
| Improve | 3 | 12.5 | 2 | 6.2 | 4 | 19.0 | 2 | 7.7 |
| Worsen | 6 | 25.0 | 3 | 9.4 | 5 | 23.8 | 3 | 11.5 |
| Total | 24 | 100.0 | 32 | 100.0 | 21 | 100.0 | 26 | 100.0 |
|  |  |  |  |  |  |  |  |  |
|  |  |  |  |  |  |  |  |  |
| **Self-care** |  |  |  |  |  |  |  |  |
| No change | 23 | 95.8 | 31 | 96.9 | 19 | 90.5 | 24 | 92.3 |
| Improve | 1 | 4.2 | 0 | 0.0 | 1 | 4.8 | 0 | 0 |
| Worsen | 0 | 0.0 | 1 | 3.1 | 1 | 4.8 | 2 | 7.7 |
| Total | 24 | 100.0 | 32 | 100. | 21 | 100.0 | 26 | 100. |
|  |  |  |  |  |  |  |  |  |
|  |  |  |  |  |  |  |  |  |
| **Usual activities** | |  |  |  |  |  |  |  |
| No change | 14 | 58.3 | 25 | 78.1 | 14 | 66.7 | 21 | 80.8 |
| Improve | 4 | 16.7 | 2 | 6.2 | 2 | 9.5 | 1 | 3.8 |
| Worsen | 6 | 25.0 | 5 | 15.6 | 5 | 23.8 | 4 | 15.4 |
| Total | 24 | 100.0 | 32 | 100.0 | 21 | 100.0 | 26 | 100.0 |
|  |  |  |  |  |  |  |  |  |
|  |  |  |  |  |  |  |  |  |
| **Pain/discomfort** | |  |  |  |  |  |  |  |
| No change | 11 | 45.8 | 15 | 46.9 | 12 | 57.1 | 11 | 42.3 |
| Improve | 4 | 16.7 | 13 | 40.6 | 4 | 19.0 | 10 | 38.5 |
| Worsen | 9 | 37.5 | 4 | 12.5 | 5 | 23.8 | 5 | 19.2 |
| Total | 24 | 100.0 | 32 | 100.0 | 21 | 100.0 | 26 | 100.0 |
|  |  |  |  |  |  |  |  |  |
|  |  |  |  |  |  |  |  |  |
| **Anxiety/depression** | |  |  |  |  |  |  |  |
| No change | 16 | 66.7 | 24 | 75.0 | 13 | 61.9 | 21 | 80.8 |
| Improve | 2 | 8.3 | 6 | 18.8 | 1 | 4.8 | 3 | 11.5 |
| Worsen | 6 | 25.0 | 2 | 6.2 | 7 | 33.3 | 2 | 7.7 |
| Total | 24 | 100.0 | 32 | 100.0 | 21 | 100.0 | 26 | 100.0 |
